# Computational characterization of domain‐segregated 3D chromatin structure and segmented DNA methylation status in carcinogenesis

**DOI:** 10.1002/1878-0261.13127

**Published:** 2021-11-09

**Authors:** Yue Xue, Ying Yang, Hao Tian, Hui Quan, Sirui Liu, Ling Zhang, Lu Yang, Haichuan Zhu, Hong Wu, Yi Qin Gao

**Affiliations:** ^1^ Beijing National Laboratory for Molecular Sciences College of Chemistry and Molecular Engineering Peking University Beijing China; ^2^ The MOE Key Laboratory of Cell Proliferation and Differentiation School of Life Sciences Peking University Beijing China; ^3^ Peking‐Tsinghua Center for Life Sciences Peking University Beijing China; ^4^ Peking University Institute of Hematology National Clinical Research Center for Hematologic Disease Peking University People’s Hospital Beijing China; ^5^ Biomedical Pioneering Innovation Center (BIOPIC) Peking University Beijing China; ^6^ Beijing Advanced Innovation Center for Genomics (ICG) Peking University Beijing China

**Keywords:** carcinogenesis, chromatin structure, DNA methylation, gene expression dysregulation

## Abstract

The high‐order chromatin structure, together with DNA methylation and other epigenetic marks, plays a vital role in gene regulation and displays abnormal status in cancer cells. Theoretical analyses are expected to provide a more unified understanding of the multi‐omics data on the large variety of samples, and hopefully a common picture of carcinogenesis. In particular, we are interested in the question of whether an underlying origin DNA sequence exists for these epigenetic alterations. The human genome consists of two types of megabase‐sized domain based on the distribution of CpG islands (CGIs) that show distinct structural, epigenetic, and transcriptional properties: CGI‐rich and CGI‐poor domains. Through an integrated analysis of chromatin structure, DNA methylation, and RNA sequencing data, we found that, in carcinogenesis, the two different types of domain display different structural changes and have an increased number of DNA methylation differences and transcriptional‐level differences, compared with in noncancer cells. We also compared the structural features among carcinogenesis, senescence, and mitosis, showing the possible connection between chromatin structure and cell state, which could affect vital cancer‐related properties. In summary, chromatin structure, DNA methylation, and gene expression, as well as their changes observed in several types of cancers, show a dependence on multiscale DNA sequence heterogeneity.

## Graphical abstract

By employing an integrated analysis of multi‐omics data, we observed that the changes in chromatin structure, DNA methylation and gene expression related to carcinogenesis are dependent on multiscale DNA sequence heterogeneity. These concerted changes that we identified in several cancer types offer a new perspective in understanding the molecular mechanisms behind cancer development.

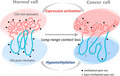

**Abbreviations**
CGI: CpG islands
CI: compartment index
F: CGI forest domain
Hi‐C: high‐throughput chromosome conformation capture
IS: insulation score
MDI: F‐P methylation difference
P: CGI prairie domain
TAD: topologically associated domain
TPM: transcripts per million
TSS: transcription start site
WGBS: whole‐genome bisulfite sequencing

## 1 Introduction

Three‐dimensional chromatin structure plays a vital role in gene regulation. The development of chromosome conformation capture [1] (3C) technology and its derived methods, such as Hi‐C [2] and ChIA‐PET [3], significantly improves our understanding of genome organization. For instance, the anchors of chromatin loops that frequently link enhancers and promoters are occupied by CCCTC‐binding factor (CTCF) and cohesin complex in most cases [4]. Such insulator structures can help maintain normal gene expression [5, 6]. For cancer, many studies have revealed that mutations of CTCF binding sites and disruptions of insulated structures could result in dysregulation of gene expression [6, 7, 8], an intrinsic property in cancer. Besides, structural variants, such as deletions, inversions, and translocations, are recurrent in multiple cancer types [9]. Previous studies identified a positive correlation between translocation frequency and spatial proximity [10]. A recent paper [11] has shown an integrative strategy to comprehensively detect these variants and captured numerous instances related to structural changes such as the fusion or loss of topologically associated domains (TADs), the median size of which is several hundred kilobases. Nevertheless, unlike early embryonic development [12] and cell differentiation [13], the overall structural changes in carcinogenesis remain to be elucidated.

Along with aberrant 3D chromatin architecture, drastic genome‐wide epigenetic changes also take place in carcinogenesis [14, 15], jointly influencing gene expression. Many studies have shed light on the stable epigenetic alterations associated with cancer cells, and DNA methylation was firstly and most widely studied [16, 17]. There are mainly two types of general DNA methylation changes in cancer cells: global hypomethylation of late‐replicating lamin‐associated domains (LADs) [18] and hypermethylation of specific CpG islands (CGIs) [19, 20]. Over ten thousands of publications reported DNA methylation changes as cancer biomarkers [21], and recently some evidences show that DNA methylation has little impact on gene expression but corresponds to chromosomal structural changes [22, 23]. However, the correlation between changes of DNA methylation and cancer development and the relationship between methylation and chromosomal structure remain largely unexplored.

In principle, both chromosomal structure and epigenetic modifications can influence gene expression. Based on Hi‐C contact map, the chromatin is divided into compartments A and B [2]. Genes are enriched in compartment A, and their expression levels are higher than those in compartment B. However, there are many questions remain unanswered, for example, what factors determine the compartment formation, what are the driving forces of compartment switch, and what are the roles of compartmentalization in cancer? Our previous study [24] showed that the compartment formation is strongly related to the genome composition. Based on the uneven distribution of CGIs, the whole genome was divided into two types of megabase‐sized domains, CGI‐rich domains (named as CGI forest domains), and CGI‐poor domains (named as CGI prairie domains). These two types of domains, differing in sequence features, show distinct epigenetic and transcriptional patterns and overlap strongly with the compartments A and B, respectively. Furthermore, the cell‐specific spatial contact and separation between these two types of domains are strongly coupled with various biological processes, such as early embryonic development [25], cell differentiation, and senescence [26]. The main goal of this study is to interrogate the sequence dependence of various carcinogenesis marks and to investigate the intrinsic mechanisms of cancer development. It was found here that forest and prairie domains behave significantly differently in carcinogenesis, including their distribution in compartments, CGI interactions, TAD formation, gene expression, and DNA methylation, which is closely associated with development stage of cancer. Besides, the methylation state of regions with low CpG density could reflect the chromatin structure. We also found that the regulation of gene expression depends on the sequence feature in a scale‐dependent manner.

## 2 Materials and methods

### 2.1 Source of methylome data

The whole‐genome bisulfite sequencing (WGBS) data of methylomes were obtained from The Cancer Genome Atlas (TCGA) [27] project and Gene Expression Omnibus, including 48 cancer samples and 17 matched adjacent samples, as well as paired cancer and normal data of 4 liver, 3 lung [28], and 2 colon cancer samples [29, 30]. The reference genome is hg19. Normal liver and lung methylomes and those of their corresponding cancer cell and cancer cell lines were downloaded from Roadmap [31] and Encode Project [32] for combinatorial analysis of histone modifications and Hi‐C contact. The description and references of the data sets are summarized in Table S1. To ensure the credibility of the analysis results, in our calculation we only use CpG sites with coverage greater than three. DNA methylation level of each CpG site was given in percentage by.

$$\beta =\frac{M}{M+U}\cdot 100\%$$

where M and U are the signal strength of methylated and unmethylated CpG, respectively.

In this work, we focus on all protein coding genes which are downloaded from GENECODE release 19 (https://www.gencodegenes.org).

### 2.2 Definition of F, P, and F‐P methylation difference (MDI)

The definition of CGI forest and CGI prairie follows our previous work [24]. Briefly, we defined and calculated critical neighboring CGI distances, longer than which CGIs are more likely to be next to each other than random. A CGI‐rich domain (CGI forest, F) was defined as a continuous DNA region longer than the critical length, and all neighboring CGI distances inside this domain are shorter than the critical length. After excluding the chromosomal unmappable and dark regions, CGI‐poor domains (CGI prairies, P) were defined as the complementary regions of forest (Table S2).

Following our previous work [24], the methylation difference in open sea between neighboring forests and prairies is defined as follows:

$$MDI_{i}=\frac{\left(q_{i}‐\frac{q_{i‐1}+q_{i+1}}{2}\right)}{\left(\frac{q_{i}+q_{i‐1}+q_{i+1}}{3}\right)}$$

where $q_{i}$, $q_{i‐1}$, and $q_{i+1}$ are the methylation level for the *i*th domain and its two flanking domains.

### 2.3 Gene function analysis

GO enrichment analysis of all the given gene clusters in this work was conducted using the R package ClusterProfiler [33]. Individual gene functions were obtained from GeneCards (https://www.genecards.org). Immune‐related genes were obtained from AmiGO2 (http://amigo.geneontology.org/amigo).

### 2.4 Definition of tissue specificity for gene

The normalized RNA‐seq data of GTEx project [34] were downloaded from Ref. [35]. The tissue specificity of gene i in tissue t was defined as.

$$s_{i}^{t}=\frac{\varepsilon_{i}^{t}‐\mu_{i}^{\mathrm{all}}}{\mu_{i}^{\mathrm{all}}}$$

where $\varepsilon_{i}^{t}$ and $\mu_{i}^{\mathrm{all}}$ are the mean expression level of gene i in tissue t and all tissues examined, respectively. A gene with a tissue specificity value greater than 2 was defined as a tissue‐specific gene.

### 2.5 Chromatin 3D structure analysis

All human Hi‐C data [36, 37, 38] in this work were normalized by ICE method at a 40‐kb resolution using the iced python package [39]. Mouse cell cycle Hi‐C data [40] were normalized at 100‐kb resolution, and the reference genome is mm9. Chromosome structural alterations for cancer cell line samples, which were identified by hic_breakfinder in researches [11, 41], were removed from Hi‐C data. Genomic locations which have no contacts with more than 99% of other locations were also deleted in all samples.

#### 2.5.1 Compartment identification

The identification of compartments A and B in 200‐kb resolution mainly followed the Lieberman–Aiden’s approach [2]. Briefly, a correlation matrix was calculated based on normalized chromosome contact matrix. Subsequent eigenvector analysis partitioned the chromosome into two spatial compartments. We further made a slight modifications according to our previous work [24], in which to eliminate the influence of the centromere, the Hi‐C matrix was disassembled into two parts, corresponding to p and q arms, and the eigenvalue decomposition was done within these two arms separately.

#### 2.5.2 Compartment index calculation

To quantify the compartmentalization degree [42], a compartment index CI_i_ for 200‐kb bin i (the same size as compartment definition) was calculated as the logarithm ratio of the average contact between this bin and all compartment A over that between this bin and all compartment B:

$$\begin{array}{l} {\text{CI}}_{i}=\ln\left(\frac{\frac{\sum_{j,j\neq i}C_{\mathrm{ij}}\delta_{j}}{N_{A}}}{\frac{\left(\sum_{j,j\neq i}C_{\mathrm{ij}}\left(1‐\delta_{j}\right)\right)}{N_{B}}}\right),\\ \delta_{j}=\left\{\begin{array}{c} 1\;\text{if bin}\;\text{j}\;\text{is in compartment A}\\ 0\;\text{if bin}\;\text{j}\;\text{is in}\;\text{compartment B} \end{array}\right. \end{array}$$

where C_ij_ is the normalized Hi‐C contact probability between bins i and j. *N*_A_ and *N*_B_ are the bin numbers of compartment A and B, respectively. And the self‐contact was excluded in this calculation. For each 200‐kb bin, a positive CI indicates it contacts more frequently with compartment A than compartment B.

#### 2.5.3 Interaction strength

The 40‐kb bin (in accordance with the resolution of Hi‐C contact matrix) was identified as a CGI bin if it harbors at least one CGI; otherwise, it was labeled as a non‐CGI bin. With the above definition, each bin could spatially contact with four categories of DNA domains: CGI in CGI‐rich domains (F‐CGI), non‐CGI in CGI‐rich domains (F‐non‐CGI), CGI in CGI‐poor domains CGI (P‐CGI), and non‐CGI in CGI‐poor domains (P‐non‐CGI). The interaction strength between bin k and one of the four types of DNA segments *R*_i_ was defined as.

$$I_{k,R_{i}}=\frac{C_{k,R_{i}}}{\sum_{i=1}^{4}C_{k,R_{i}}}$$

where *R*_i_ is a vector consisting of the bins belonging to part i, *C*_k, Ri_ is the summation of all contact probabilities between bin k and *R*_i_. In this calculation, we deleted the self‐contact elements.

#### 2.5.4 Contact probability and segregation factor as functions of genomic distance

The segregation factor was calculated as the ratio between contact probabilities of DNA domains of the same (F with F, or P with P) and different genome types (F with P), reflecting the extent of forest or prairie segregation. To identify contact loss in cancer cell line, we first calculated the average contact probability at the particular range of genomic distance for each bin for both cancer and its corresponding normal tissue. If this contact probability was higher than average level of all bins in normal cells but lower than average in cancer cells, then this bin was considered as contact loss.

#### 2.5.5 Definition of insulation score (IS)

For two neighboring regions *A*_1_ and *A*_2_, the insulation score was defined as in Ref. [43],

$$\text{IS}=\text{log}\left(1+\frac{a_{1}}{b}+\frac{a_{2}}{b}\right)$$

where a_1_, a_2_, and b represent the mean contact probability inside *A*_1_ and *A*_2_ that between them, respectively. *A*_1_ and *A*_2_ can represent not only the forest and prairie domains but also any two windows with the same size.

### 2.6 Process of RNA‐seq data

We downloaded counts formatted files from TCGA project for all available RNA sequencing data of cancer and matched normal samples, and converted them to TPM (transcripts per million) format. The expression fold change in a given gene in carcinogenesis was defined as follows:

fold change = $\log_{2}\left(\frac{{\text{TPM}}_{\text{cancer}}+1}{{\text{TPM}}_{\text{normal}}+1}\right)$

where TPM_cancer_ and TPM_normal_ of a gene represent average TPM in all normal and cancer samples, respectively.

Differential gene expression analysis was performed by R/Bioconductor package ‘DESeq2’ [44]. Significantly up expressed genes were defined as *P*‐value < 0.05 and fold change > 1, and significantly downexpressed genes were defined as *P*‐value < 0.05 and fold change < −1 calculated by DESeq2.

## 3 Results

### 3.1 DNA methylation changes coupled to cancer development

**Fig. 1:**
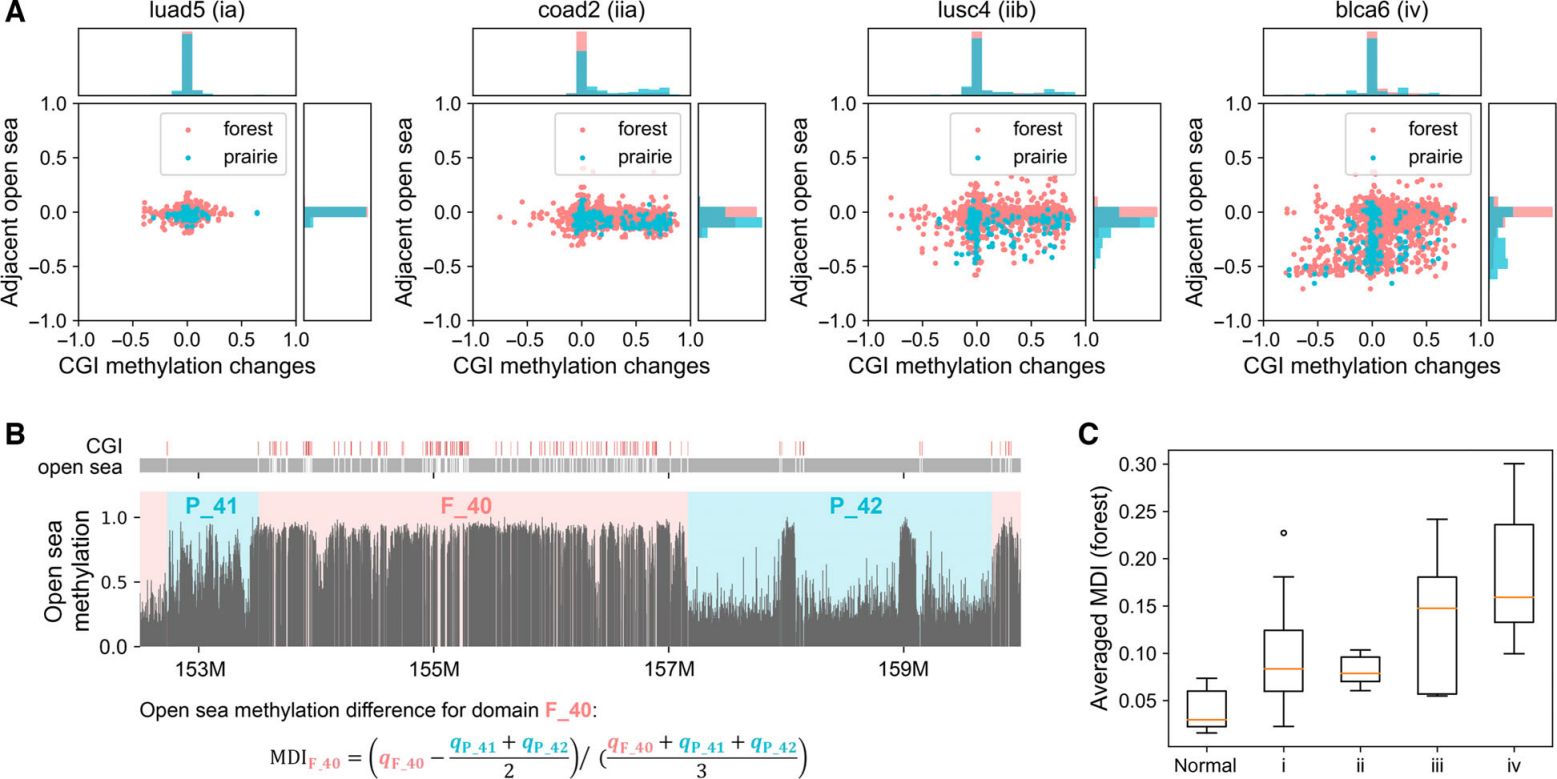
Methylation changes in carcinogenesis. (A) Scatter plots for changes in methylation level in CGIs and open seas. Each dot represents the methylation level changes in a CGI (x‐axis) on chromosome 1 and its adjacent open sea (y‐axis) changing from adjacent normal samples to corresponding cancer samples. The probability density distribution of CGI and open sea methylation‐level changes is shown on the top and right sides of the figure, respectively. The cancer stages are given next to the sample names. (B) The methylation levels for regions in open sea (chromosome 1 in sample blca_t6) and the calculation of MDI (F‐P methylation difference). Specifically, q represents the average open sea methylation level for a F or P domain. (C) The averaged MDIs of all forest domains in normal samples and cancer samples at different stages. Data are represented as boxplots where the box extends from the lower to upper quartile values of the data, with a line at the median. The whiskers extend from the box to show the range of the data. The upper and lower whisker extends no further than 1.5 × IQR from the upper and lower edges of the box, respectively (IQR is the interquartile range). The dot represents data outlier.

Based on the analysis of the WGBS data of 17 patients (including 9 types of cancer) and their adjacent normal tissues, it was found that, consistent with previous studies, the changes in DNA methylation from normal cells to cancer have two general characteristics: hypomethylation in the open sea (regions beyond 4000 base pairs upstream and downstream of CGI) [45] and hypermethylation in a subset of CGIs. Moreover, the extent of methylation changes appears to correlate with the stage of cancer development. CGI hypermethylation can usually occur at early stages of cancer development (stage I and stage II) when hypomethylation of open seas is relatively weak or even not significant. As the cancer stage progresses, the open seas become more frequently and deeply hypomethylated. Similar trends of methylation changes are observed among a variety types of cancers, indicating the similarity in the development of different cancers or even potential common causes (Fig. 1A and Fig. S1).

#### 3.1.1 Domain‐dependent hypomethylation of open sea reflects the development of cancer

In most normal tissues, CpGs are mainly methylated in the open sea and the average open sea CpG methylation level in prairies (P) is slightly lower than that in forests (F). In carcinogenesis, open sea CpGs in prairies are more significantly hypomethylated than forests, leading to the increased methylation difference between forests and prairies (*P*‐value = 5.4 × 10^−6^ by Welch’s unequal variance *t*‐test; Fig. 1B). The hypomethylation of the prairies gives rise to most of the PMDs observed earlier [46] (Fig. S2A). To quantify the difference between the open sea methylation levels of F and P domains, we calculated the averaged F–P methylation differences (MDI; Fig. 1B, see Methods) for each sample and found that in normal tissues, the averaged MDIs for forests are always positive and that for prairies, negative, suggesting that the open sea methylation level of forests is in general higher than that of adjacent prairies. In cancer cells, averaged MDIs for forests become larger than their adjacent normal cells for almost every cancer sample (Fig. S2B). Remarkably, the averaged MDIs of forests generally increase with the aggravation of cancer, implying that the open sea methylation difference between forest and prairie domains does reflect the stage of cancer development (Fig. 1C and Fig. S2C).

Furthermore, we found that the probability of hypomethylation increases with the decrease in CpG density in open seas of both forest and prairie domains (Fig. S2D). In addition, the methylation level of prairie open seas is lower than that in forests even when they have the same CpG density. Such a result suggests that the prairie domains undergo more severe hypomethylation during carcinogenesis than the forest domains, suggesting that not only the local low CpG density but also the surrounding sequence environment influences the methylation level of an open sea region.

### 3.2 General features of cancer chromatin structure

#### 3.2.1 Intrinsic sequence preference for compartment formation

The chromatin structural differences between somatic and cancer samples were investigated in the following. We used A549 cancerous lung cell line, Panc1 pancreatic cancer cell line, and HepG2 liver cancer cell line as representative cancer samples and compared them with somatic lung, pancreas, and liver samples. Firstly, the chromosome structural variants [11, 41] (SVs, including translocations, duplications, and deletions) were removed from cancer cell line samples, and outliers in contact matrix were also removed from all samples (see Methods). Chromatin compartments in cancer and somatic samples differ in extent of segregation. We used compartment vector components to divide the chromosome into compartments A and B (Table S3).

**Fig. 2:**
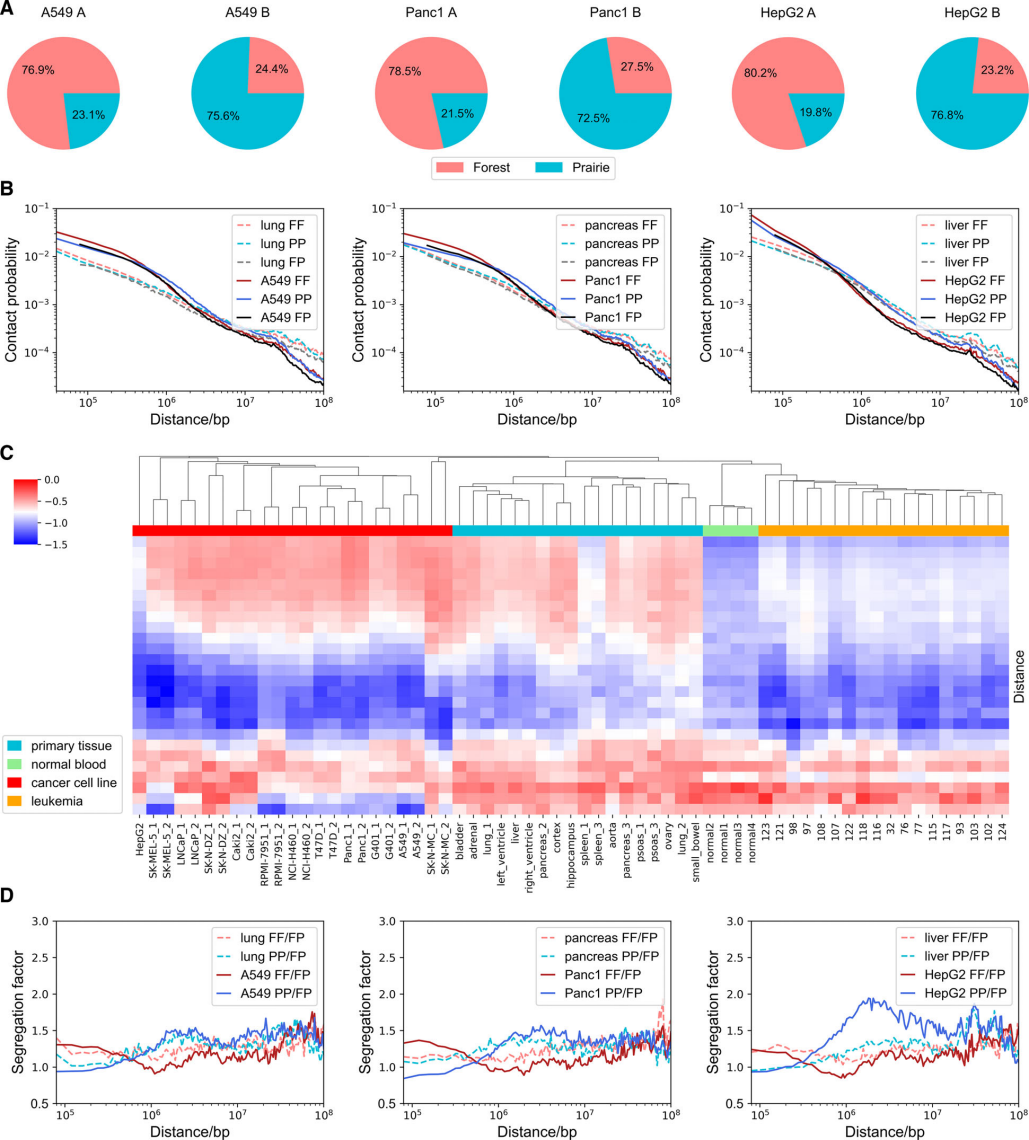
General chromatin architecture in cancer cell lines. (A) The proportion of forest and prairie sequences in compartments A and B for A549, Panc1, and HepG2. (B) The contact probability between forests and forests (FF), prairies and prairies (PP), forests and prairies (FP) at varied genomic distances for lung and A549, pancreas and Panc1, liver and HepG2 (chromosome 1 is used as an example). (C) Hierarchical clustering for the decay slopes of the PP contact probability at various genomic distances. From upper to lower are slopes from short‐range to long‐range genomic distances. Euclidean distance is used as the distance metric, and nearest point algorithm is applied for hierarchical clustering. (D) The segregation factor at varied genomic distances for lung and A549, pancreas and Panc1, liver and HepG2 (chromosome 1 is used as an example).

Compartment formation is seen to largely follow DNA sequence characteristics, separating forests from prairies (Fig. 2A and Fig. S3A). For both normal and cancer samples, compartment index (see Methods) of FB (forest domains in compartment B) is larger than PB, and at the same time, the compartment index of FA is larger than PA (Fig. S3B). These results indicate that the structure environment for PA (FB) is not as open (compact) as common FA (PB), contributing from their own sequence environment. Changing from normal tissue to cancer cell line, a subset of forests switch from compartments A to B and their CpG densities are lower than those of forests conserved in compartment A (Fig. S3C). These observations indicate that in cancer cells, compartment B, which constitutes mainly prairie domains, tends to expand to forests of low CpG densities. Tissue‐specific genes also show preferential distribution in the compartment switch. Prairies are enriched with tissue‐specific genes of various tissues as shown in our earlier study [24]. In normal cells, genes specific to other tissues (complementary tissue‐specific genes) are repressed and more likely located in compartment B. In cancer cells, a large proportion of prairie genes switching from compartments B to A are complementary tissue‐specific genes (Fig. S3D), suggesting the loss of cell identity in cancer cells. We next used the averaged compartment vector component $\bar{V}$ to quantify the DNA sequence preference of compartments (Table S4). A high $\bar{V}$ of a DNA domain implies that it has a high tendency to reside in compartment A. These data show that CGI domains and forests generally possess higher $\bar{V}$ than non‐CGI domains and prairies, respectively, for both normal and cancer samples. CGI domains in forest (F‐CGI) are often stable in compartmentalization, whereas CGI domains in prairies (P‐CGI) tend to shift to compartment B, again demonstrating a DNA sequence preference in the change in compartment segregation. The implication and biological function of such changes in chromatin compartmentalization will be analyzed as follows.

#### 3.2.2 Overall chromatin architecture in cancer

Next, we tried to investigate the chromatin structural changes at a broad range of genomic length scales. We first analyzed the contact probability changes at varied genomic distances (see Methods) and observed that overall the contact probability decays faster as a function of genomic distance for cancer cells than normal cells, which indicates a loss of long‐range spatial contacts in carcinogenesis (Fig. S3E). Further investigation revealed that the F‐P contact is weaker than F‐F and P‐P contacts at nearly all sequential distances, indicating the overall separation between these CGI‐rich and CGI‐poor domains (Fig. 2B). The contact probability calculated for the normal lung cell decays following almost a single power‐law in the genomic distance range of hundreds of kilo‐ to several mega‐bases (slope = −0.76 and −0.69 for F‐F and P‐P contacts, respectively), indicating a relatively uniform contact probability scaling property for normal tissues. In contrast, the cancer samples exhibit a scale separation in contact probability decay curve, with a slower decay for both F‐F (slope = −0.56) and P‐P (slope = −0.57) contacts than corresponding somatic samples at distances shorter than 400 kb (F‐F contact) and 800 kb (P‐P contact), and a steeper decay (slope = −1.38 and −1.26 for F‐F and P‐P contacts, respectively) at large distances (Fig. S3E). To investigate the universality of the spatial contact differences between normal and cancer cells, we calculated the decay slopes of P‐P contact probability at various genomic distances for 18 primary normal samples (14 types of tissues), 23 cancer cell line samples (10 types), 4 normal blood samples, and 18 leukemia samples. Hierarchical clustering results show the distinct spatial contact pattern among normal tissues, cancer cell lines, and leukemias, as well as the high similarity within each type of samples, implying that the 3D chromosome structure is an important variable to investigate in carcinogenesis (Fig. 2C).

To further compare the relative contact strength of forests and prairies at varied distances, we defined and calculated the segregation factor (see Methods). A high segregation factor for a DNA segment (e.g., of 40‐kb) indicates that it prefers to contact with domains of the same type over those of a different type at that given genomic distance. For both normal and cancer samples, the segregation factor is almost always greater than 1 at all genomic distances, suggesting an overall F‐P domain separation (Fig. 2D). At short distances (less than 500 kb), the segregation factor is higher for forests than for prairies. As the genomic distance increases, its value decreases for forests and increases for prairies. Such a trend is more obviously seen in the cancer cell lines than in the normal samples. These observations indicate that forest domains have strong contacts at short distances, especially between DNA segments within the same forest. Spatial contacts between forest domains are weak at a genomic distance of  ~1Mb. In contrast, contacts between nearby prairie DNAs are weak but when the genomic distance increases to millions of kilobases, prairie domains tend to interact frequently, indicating the loss of local contacts in the expense of long‐range (intra‐ and inter‐domain) prairie contacts. The repetitive elements [47] are differently distributed in the forest and prairie domains, and short interspersed nuclear elements (SINEs) are enriched in forest domains whereas long interspersed nuclear elements (LINEs) are more enriched in prairie domains (Fig. S3F, S3G). According to the length of the SINEs (or LINEs) for each 40‐kb domain, 80.3% of high SINE density domains are forest domains and 71.7% of low SINE density domains are prairie domains, 68.8% of low LINE density domains are forest domains, and 60.3% of high LINE density domains are prairie domains. Domains with high and low densities of SINEs (or LINEs) also show similar spatial separation in carcinogenesis, although to a less extent compared to that between forest and prairie domains (Fig. S3H, S3I).

Intriguingly, a number of forest genes lose contact with other forest domains at genomic distances ranging from 600 K to 2 m in cancer cells, contributing to the weakened segregation factor for forest domains at  ~1 m. The chromatin interactions detected by Fit‐Hi‐C [48] clearly show the contact loss in these regions (Fig. S4A). These genes are heavily shared among A549, Panc1, and HepG2 (all *P*‐values < 10^−150^ by Fisher’s exact test between A549 and Panc1, between A549 and HepG2, between Panc1 and HepG2), and many are related to the immune process (Fig. S4B, S4C, S4D). For instance, 29.6% of the genes related to antigen processing and presentation and 26.1% of immune system genes are involved in the F‐F contact loss in A549 cancer cell line. In Panc1 cell line, the proportions are 27.9% and 27.6%, and in HepG2 cell line, the proportions are 22.5% and 24.8% (Table S5). For example, a forest gene RELA, which is a proto‐oncogene and subunit of NF‐κB, is found to lose contact with forest domains in three types of samples. Dysregulation of NF‐κB is a hallmark of cancer and can promote genetic and epigenetic alterations, change cellular metabolism, directly and indirectly control inflammation, cancer cell proliferation and survival, epithelial‐to‐mesenchymal transition, invasion, angiogenesis, and metastasis [49]. Commonly affected genes also include kinesins, the misregulation of which are involved in cancer pathogenesis, such as uncontrolled cell growth and metastasis [50, 51]. At the same time, a group of growth factors are also involved in this chromosome structure change. How these changes contribute to cancer initiation and development remains to be further investigated.

**Fig. 3:**
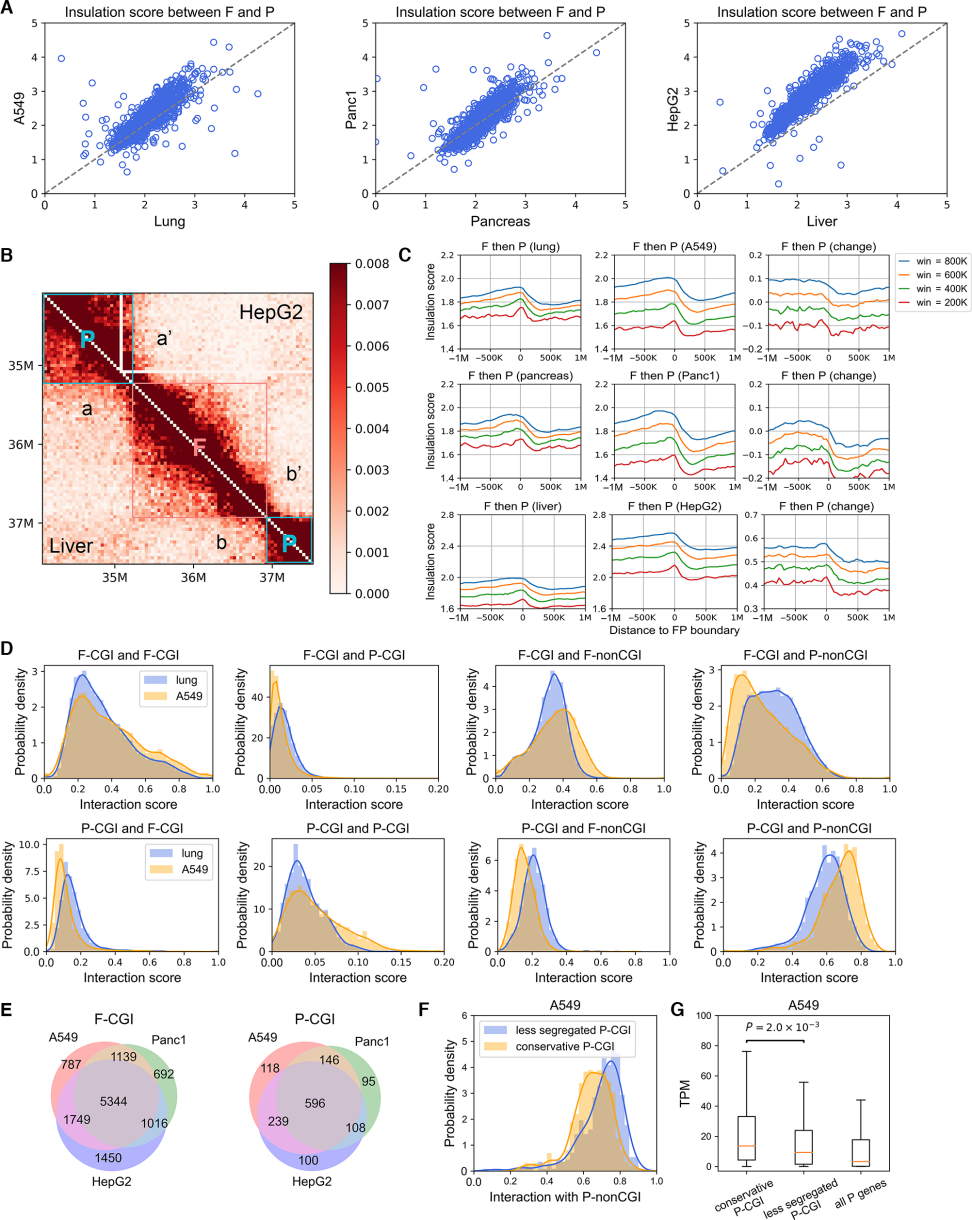
Domain insulation and CGI aggregation in carcinogenesis. (A) The insulation scores between adjacent forest domain and prairie domain in lung and A549, in pancreas and Panc1, in liver and HepG2. (B) The contact probability matrix of liver (lower triangular matrix) and HepG2 (upper triangular matrix) (chromosome 1). Forest and prairie domains are marked with square frames. The contact probabilities in a’ and b’ are lower than a and b regions, showing an increased insulation between forest and prairie in HepG2. (C) The insulation scores for 40‐kb beads around F‐P boundary at different window sizes in normal cells (left), cancer cells lines (middle), and the differences between them (right). The data are aligned so that the forest domains are positioned to the left of the boundary (value 0). (D) The interaction scores between F‐CGI (top row) or P‐CGI (bottom row) and the four types of domains (F‐CGI, F‐non‐CGI, P‐CGI, and P‐non‐CGI) in normal lung and A549. All *P*‐values < 10^−90^ by *t*‐test). (E) The overlap of aggregated CGIs among A549, Panc1, and HepG2. (F) The probability density of interactions with P‐non‐CGI for conservative P‐CGIs and P‐CGIs becoming less segregated in A549. *P*‐values = 1.3 × 10^−12^ by Welch’s unequal variance *t*‐test. (G) The expression level for conservative P‐CGI genes, less segregated P‐CGI genes and all prairie genes in A549. Expression level for each gene is calculated by averaging TPM (transcripts per million) over all LUAD cancer samples. Data are represented as boxplots, and *P*‐values are calculated by Welch’s unequal variance *t*‐test.

We also used the insulation score (IS, see Methods) to explore the structure changes in carcinogenesis. From the perspective of the domain level, the IS between adjacent forests and prairies was significantly larger in tumor than in normal cells (*P*‐value = 4.12 × 10^−83^, 6.28 × 10^−10^ and < 10^−300^ by *t*‐test for lung, pancreas, and liver, respectively; Fig. 3A), again hinting the formation of a structure with forest and prairie domains significantly separated (Fig. 3B). We next investigated the spatial insulation around forest, prairie domain boundaries at varied window sizes (Fig. 3C). For both normal and cancer cell lines, the insulation score is generally higher for forest than prairie domains, indicating more local interactions within forest, accordant with our finding that forests to be mainly composed of type A whereas prairies, type B [24]. Furthermore, for lung and pancreas, the IS in both forest and prairie in tumor is smaller than that in normal cells at small window sizes (e.g., 200 kb), indicating a more homogeneous distribution of contact around the main diagonal of Hi‐C matrix. As the window size increases, forests and prairies display distinctly different insulation behaviors. For forests, the cancer IS values become larger than the corresponding values in normal tissues when the window size > ~ 500 kb, indicating that the interactions in forest domains becomes increasingly dominated by local contacts. In contrast, the IS values in prairie domains are smaller in cancer samples compared with normal cells at larger range of window sizes than that in forest. The spatial contact in HepG2 is more locally dominant than A549 and Panc1; therefore, the IS values increase at all window sizes we examined. The extent of increasing insulation is always higher for forest domains than prairie domains, which could be observed in all three types of cancers.

#### 3.2.3 CGI aggregation strengthens in carcinogenesis

We next focused on the local chromatin structure and investigated the 3D contact changes of CGIs and their underlying biological implications. We first calculated the interaction strengths (see Methods) for both normal cells and cancer cell lines. Taking lung as an example, from normal to cancer, contacts between the same genome types (F‐CGI and F‐CGI, F‐CGI and F‐non‐CGI, P‐CGI and P‐CGI, P‐CGI and P‐non‐CGI) increase, accompanied by the reduced contacts between different genome types (F‐CGI and P‐CGI, F‐CGI and P‐non‐CGI, P‐CGI and F‐CGI, P‐CGI and F‐non‐CGI) (Fig. 3D). These results clearly show the enhanced spatial segregation between forests and prairies in cancer cell lines. Non‐CGI DNA regions also display a similar tendency (Fig. S5A).

Similar results are also obtained for pancreas cancer (Fig. S5B, S5C) and liver cancer (Fig. S5D, S5E). Intriguingly, F‐CGIs and P‐CGIs forming strong contact with their same types (between F‐CGIs and between P‐CGIs) in cancer are highly conserved among lung, pancreas, and liver (Fig. 3E). For the convenience of discussion, we hereinafter name these common CGIs conservative CGIs. Notably, in cancer, compared with less segregated P‐CGIs, the conservative P‐CGIs show significantly lower contact probability with P‐non‐CGI regions (*P*‐values = 1.3 × 10^−12^, 1.2 × 10^−8^ and 5.1 × 10^−44^ by Welch’s unequal variance *t*‐test in A549, Panc1, and HepG2, respectively; Fig. 3F and Fig. S5F), the less active chromatin domains. This observation indicates that the aggregation of P‐CGI during carcinogenesis may result in a more open and active environment (although within compartment B) which attributes to the change of gene expression level (Fig. 3G and Fig. S5G).

Gene activation related to CGI aggregation is found to closely connect to cancer development. We found that upregulated forest genes harboring conservative CGIs in three kinds of cancer cells are all associated with cell cycle and glycosylation. The latter affects cell communications and interactions, known to play vital roles in cancer development and progression [52]. These genes are also enriched in functions such as embryonic organ morphogenesis, in line with the relationship between carcinogenesis and early embryo development [53], and are worthy of further investigations. In the conservative prairie regions, functions of upregulated genes in three kinds of cancer cells are all related to development and Wnt signaling pathway, the latter being linked to cancer and playing important roles in regulating development [54]. Such genes found in the analyses of liver samples also act on epithelial‐to‐mesenchymal transition, contributing to the cell growth and invasiveness in carcinogenesis [55]. These analyses thus suggest that the spatial aggregation of CGIs and functional changes is likely correlated in tumorigenesis (Table S6). The differences between cancer cell lines and normal cells in terms of DNA contacts, segregation factors, and insulation scores provide a consistent picture for chromatin structural change in carcinogenesis. Interestingly, the changes in contacts between forest domains and those between prairie domains occur at genomic distances corresponding to their TAD sizes [24, 42], respectively, indicating the improved formation of TADs and reduced inter‐TAD contacts in both forests and prairies.

#### 3.2.4 Chromatin structure in cell cycle and different cell states

**Fig. 4:**
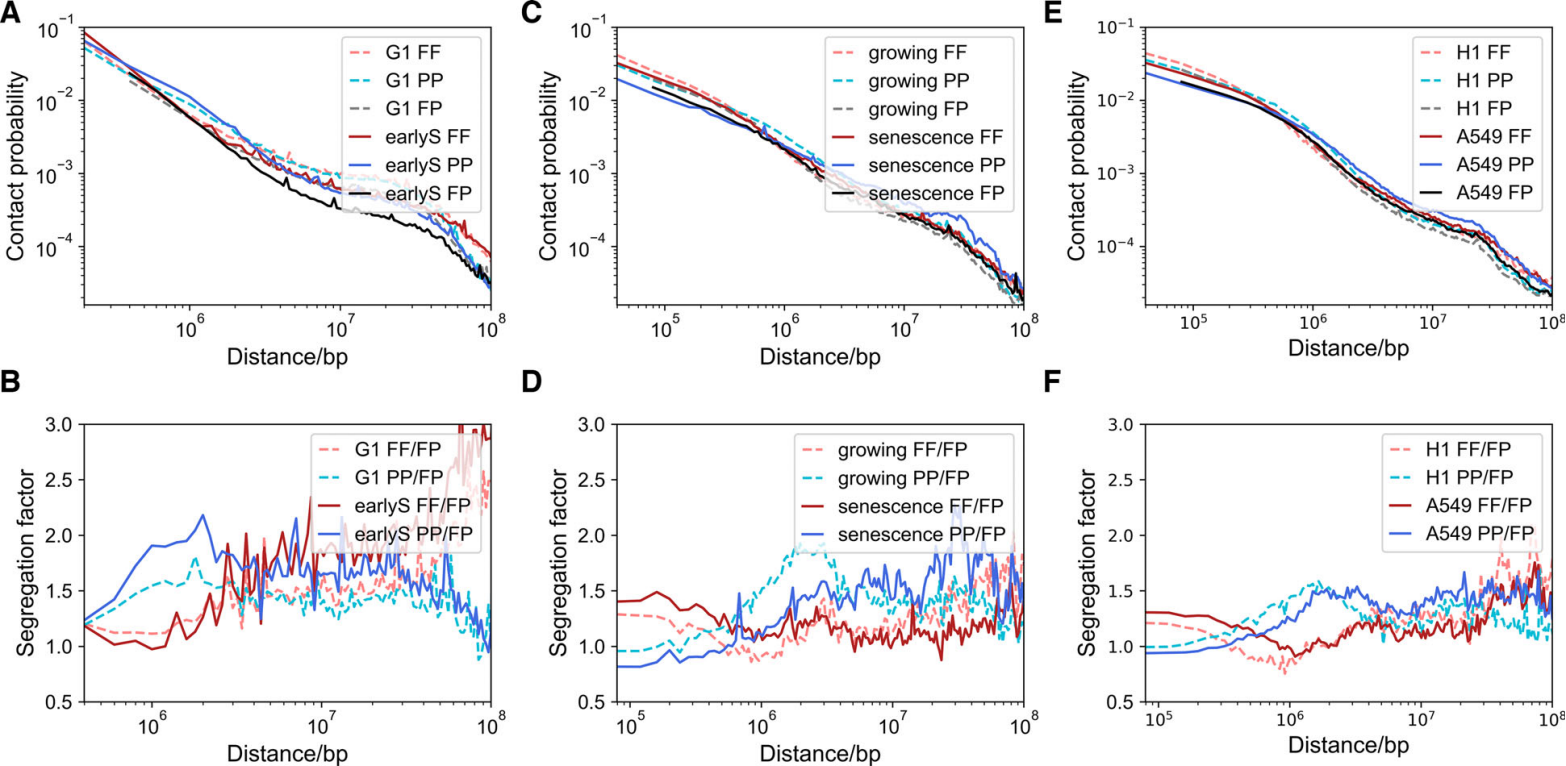
The Contact probability and segregation factor at varied genomic distances on chromosome 1. (A) (B) Cells at G1 and early S‐stage in mouse cell cycle. (C) (D) Growing cells and senescence cells. (E) (F) H1 and A549.

To examine the possible relation between cancer cells and cell division, we analyzed the chromatin structure at different stages in cell cycle for mouse, including G1, early S, late S to G2, and pre‐M. Interestingly, we observed an enhanced spatial separation between forest and prairie when the cell changes from G1‐ to early S‐stage, similar to what is observed in carcinogenesis (Fig. 4A,B). Cells at early S‐stage possess lower F‐F contact compared to F‐P at genomic distances around 1 m, but the affected genes are different from those affected in cancer cells by a similar structural chromatin change. The genes in the former process are significantly related to cell division, such as nucleosome assembly and DNA packaging (Fig. S4E). Cells from G1 to early S exhibit higher P‐P contact than F‐P at genomic distances around several million bases which is also similar to cancer cells. At large genomic distances, the chromosome structures of S‐stage cells are distinctly different from cancer cell lines. Forest domains in S‐stage cells are seen to highly spatially segregate, consistent to a clustering of the early‐replicating domain [56, 57]. On the other hand, the long‐range P domain aggregation is much weaker in S‐stage cells than in cancer cells. These observations further suggest that the chromatin structure change correlates with the realization and regulation of biological functions in processes varying from carcinogenesis to mitosis, which presumably occur at very different time scales. The similarity between structure changes (at the Mbp scale) of the two processes also suggests a possible role of cell division in cancer development.

Cell senescence is also known to be highly influenced by cell cycles. Senescence and carcinogenesis are mutually exclusive in most cases, although they can be induced by the same factors [58, 59]. Interesting similarities do exist between cancer cell and senescence cell chromatin structures, such as enhanced long‐range interactions, spatial segregation for repressive regions, analogous trend of hypomethylation of open sea (Fig. 4C,D). The similar trend of increased domain segregation in both tumorigenesis and senescence suggests a common driving force shared by them, possibly related to cell divisions. On the other hand, differences can also be observed between them. Compared to growing cells, senescent cells lose and cancer cell lines gain local contacts for both forests and prairies. A higher portion of long‐range chromatin contacts (especially that between forests and prairies) retained in the senescent than in the cancer cells. This latter difference may relate to cell identity retention, which is also a crucial difference between the highly and lowly differentiated cancer cells.

Furthermore, important similarities were also identified between early embryo development and carcinogenesis with respect to epigenetic regulation, gene expression, protein profiling, and other important biological behaviors [53]. From the chromatin structure point of view, short‐range contact gains in the sacrifice of long‐range ones are seen in both cancer cells and H1 (human embryonic stem cell line), in comparison with highly differentiated cells (Fig. 4E,F). The former two are both characterized by high segregation factors at short genomic distances for forests and at long distances for prairies, although forests segregate more at short distances and prairies tend to cluster at longer distances in cancer cell lines than in H1.

### 3.3 Relationship between DNA methylation and chromosomal structure

#### 3.3.1 CpG density dependence for DNA accessibility and methylation

It is well known that the unmethylated CGI is in general free of nucleosomes and more accessible to the transcription factors compared with methylated CGI and other genomic regions. In an earlier study, we showed that DNA methylation of the open sea reflects to the chromatin 3D structure. All these results suggest the importance of CpG density on CpG methylation and the openness of chromatin. Therefore, we divided DNA into four groups (Groups I, II, III, and IV) according to their CpG density per thousand base pairs [(2.0%, 20.1%), (1.0%, 2.0%), (0.5%, 1.0%), and (0, 0.5%), respectively]. (Beads located in CGIs mostly belongs to Group I as the minimum of their CpG density are 2.4%.) We then analyzed DNase I hypersensitivity and corresponding methylation data for liver and lung cancer cell lines (HepG2 and A549, respectively), as well as for somatic normal tissues with data available.

**Fig. 5:**
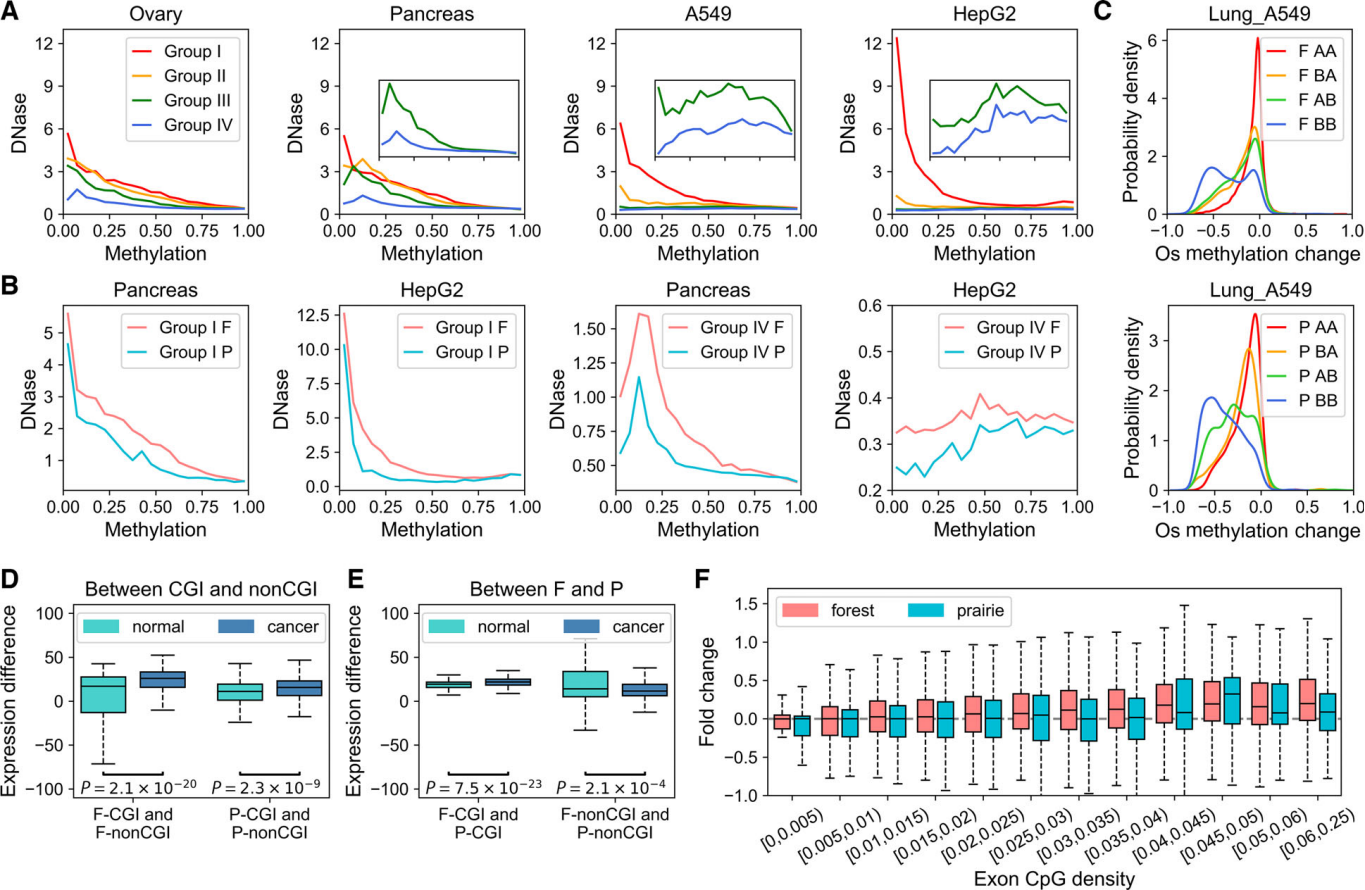
Association between methylation and chromosome structure and gene regulation. (A) Average DNase signal at various methylation levels. CpG density and CpG methylation level are calculated at a 1‐kb resolution. Bins are divided into Groups I, II, III, or IV according to their CpG density [2.0%, 20.1%], [1.0%, 2.0%], [0.5%, 1.0%], or [0, 0.5%], respectively. The last two groups (shown in green and blue lines) are magnified in the inset panels. Beads located on forest or prairie domains are shown separately in (B). (C) The probability density of open sea methylation changes for domains in forests (top) and prairies (bottom). (D) Boxplots for the average expression (TPM) differences between F‐CGI and F‐non‐CGI genes (left), between P‐CGI and P‐non‐CGI genes (right) for each normal and cancer sample. *P*‐values are calculated by Welch’s unequal variance *t*‐test. (E) Boxplots for the average expression (TPM) differences between F‐CGI and P‐CGI genes (left), between F‐non‐CGI and P‐noCGI genes (right) for each normal and cancer sample. *P*‐values are calculated by Welch’s unequal variance *t*‐test. (F) The expression fold changes in carcinogenesis (see Methods) for genes with various exon CpG density.

In normal cells, DNase I hypersensitivity of Group IV is slightly lower than other groups. In general, for normal samples DNase I hypersensitivity decreases slowly with the increase in CpG methylation level regardless of CpG density. However, in cancer cells, regions with high CpG densities and low methylation levels are much more accessible than other regions, and the DNase I hypersensitivity decreases to nearly 0 when the CpG density is lower than 0.02 or the methylation level is higher than 0.5 (Fig. 5A and Fig. S6).

Notably, with the decrease in CpG density, DNase I hypersensitivity and methylation gradually switch from being negatively to positively correlated in tumor cells, indicating that for genomic regions of very low CpG densities, higher methylation levels could reflect their higher chromatin openness. We also found that DNase I hypersensitivity of forests is constantly higher than that of prairies for any given CpG density and methylation level, and in both normal and cancer cells, consistent with the forest being in a more open and active environment [24]. Remarkably, the DNase I hypersensitivity for prairies decreases more quickly than that for forests with the decreasing of CpG density in cancer cells. Therefore, a positive correlation between DNase I hypersensitivity and methylation level persists in a larger DNA density range in prairies than that in forests (Fig. 5B and Fig. S6).

Furthermore, we controlled the GC content for each CpG density group to exclude the effect of GC bias [60] and similar correlations are observed (Fig. S7A, S7B). We also analyzed NoMe‐seq data for normal human mammary epithelial cells (HMEC) and breast cancer cell line (MCF7) [61] and found a positive correlation between methylation and DNA accessibility (whether occupied by nucleosomes) for regions with low CpG density, especially in cancer cell line (Fig. S7C). These validations indicate the correlations between DNA methylation and accessibility do exist.

#### 3.3.2 Methylation of open sea correlated to chromatin structure

To further examine the relationship between chromosomal structure and open sea methylation, we divided the genome into four groups: regions switch from compartment A to compartment B in tumorigenesis (AB), regions switch from compartment B to compartment A (BA), and those remain as A (AA) or B (BB) in both normal and tumor cells. The methylation level of AA regions remains largely unchanged while BB regions undergo the strongest demethylation, indicating that the genomic silent regions are more likely to be demethylated. Furthermore, AB regions are demethylated to a larger extent than BA, indicating that open sea demethylation tends to occur in the repressed domains of cancer cells rather than those of normal cells (Fig. 5C). It was reported that the DNA methylation could regulate the 3D chromatin structure, such as the methylation of CTCF binding sites [62, 63]. Here, we found that the methylation state of low CpG density loci correlates with the chromatin structure, which suggests a possible role for the chromatin structure in regulating the DNA methylation. The biological function of the methylation level and their interplays with chromatin structure remain to be further investigated.

There are several possible reasons behind hypomethylation and its preference to occur on prairies over forests. It was reported that CpG loci with multiple CpG sites in the surroundings are more efficiency methylated by DNMT1 [64], indicating that the local sequence feature partly contributes to the change in methylation. However, the sequence property in the large scale (forest or prairie) is also likely to affect DNA methylation. In fact, prairies tend to undergo more drastic hypomethylation than forest regions even when they have the same local CpG density (Fig. S2D). We also examined the sequence environment effects on solo‐WCGW (‘solo’ refers to the CpGs with no neighboring CpGs and ‘W’ indicates A or T nucleotide), which is reported to be the most hypomethylation‐prone sites in carcinogenesis [23] (Fig. S8). Notably, solo‐WCGWs located in prairies also have a lower methylation level in normal cells and undergo more drastic demethylation in carcinogenesis compared with those in forests, further illustrating the importance of the sequence environment.

A possible explanation for the above observations is that cancer cells undergo more frequent cell cycles than normal cells, resulting in insufficient methylation and thus a global hypomethylation. It was reported [65, 66] that for mitotic maintenance of DNA methylation, there is a global delay after replication, namely replication‐uncoupled maintenance. Due to the enlarged differences in domain structural properties and resulted different accessibility of forests and prairies, this hypomethylation is more likely to occur in the latter, enlarging the methylation difference between them. Such a mechanism is also consistent with previous findings of hypomethylation in aging cells, as well as the observation on the extent of hypomethylation being proportional to the replication timing of the regions and the cell division rate of a tissue [23, 67]. In turn, their larger methylation difference is expected to affect the contact between forests and prairies.

### 3.4 Gene expression in carcinogenesis

Next, we examined whether the change in gene expression in carcinogenesis also shows a DNA sequence dependence. We first obtained 675 pairs of transcriptome (cancerous versus adjacent normal tissues) from TCGA and compared their averaged transcription levels in CGI and non‐CGI regions and in forests and prairies (Fig. S9A). In normal cells, CGI genes (genes with CGIs on their promoter or body, see Methods) are on average more highly expressed than non‐CGI genes, and no matter they are located in forest or prairie domains. At the same time, the mean expression levels of forest genes are constantly higher than prairie genes for both CGI and non‐CGI genes. We then calculated the average expression‐level difference between CGI genes and non‐CGI genes for each sample and found these differences become enlarged in cancer cells (Fig. 5D) and the expression difference between F‐CGI and P‐CGI also increases in cancer cells (Fig. 5E). These results show that CGI/forest genes are more likely to be upregulated in carcinogenesis compared with non‐CGI/prairie genes.

To obtain more details, we also investigated the correlation between expression changes in carcinogenesis and the CpG density of gene exon and found that genes with low CpG density tend to downexpressed in cancer cells, especially for prairie genes (Fig. 5F). In general, genes with higher CpG density are more likely to be upregulated in carcinogenesis (since there are very few prairie genes of exon CpG density larger than 0.03, their expression fluctuates in high‐density groups (Fig. S9B)), showing positive correlation between expression fold change in carcinogenesis and CpG density around transcription start sites (TSSs) (Fig. S9C). At the same time, such an expression increase is practically always higher for forest genes than prairie genes of the same CpG density. When we divide all normal samples into two random groups, such changes in carcinogenesis are then not observed (Fig. S9D). The correlation between CpG density and expression fold change remains even when the genes are divided into subgroups according to their GC content (Fig. S9E), showing that the sequence property of not only the different components of the genes but also their surrounding sequences (especially, whether they reside in forest or prairie domains) can have a significant influence on their transcription activity, as well as alternation in expression in cancer development.

We also investigated the function of genes which are differentially expressed (performed by DESeq2) in multitypes of cancer cells. We analyzed 14 types of cancer (BLCA, BRCA, COAD, HNSC, KICH, KIRC, KIRP, LIHC, LUAD, LUSC, PRAD, STAD, THCA, and UCEC), each of which has more than 10 pairs of tumor and matched normal samples. Commonly differentially expressed genes are defined as their expression significantly increase or decrease in more than 7 types of cancer. F‐genes commonly upregulated are enriched in nuclear division, DNA replication, and positive regulation of cell cycle, which are consistent with the properties of tumor (Fig. S9F). Downexpressed F‐genes are closely related to muscle system processes, which may be related to alterations in the cell structure and further lead to altered deformability and cell adhesion [68]. In addition, the expression level of tissue‐specific genes is dysregulated in cancer cells. We used CIBERSORTx [69] to estimate the cell type abundances for bulk transcriptomes of LUAD and LUSC (Fig. S9G) and obtained cell type‐specific expression profiles. We found that after ruling out immune and stromal subpopulations from both normal and cancer samples, the expression of tissue‐specific genes tends to decrease in carcinogenesis while genes highly expressed in other but not the lung (complementary tissue‐specific genes) tend to increase in expression (Fig. S9H, S9I), calling for an investigation on a possible association of this gene expression‐level change with the metastasis of cancer.

## 4 Discussion

**Fig. 6:**
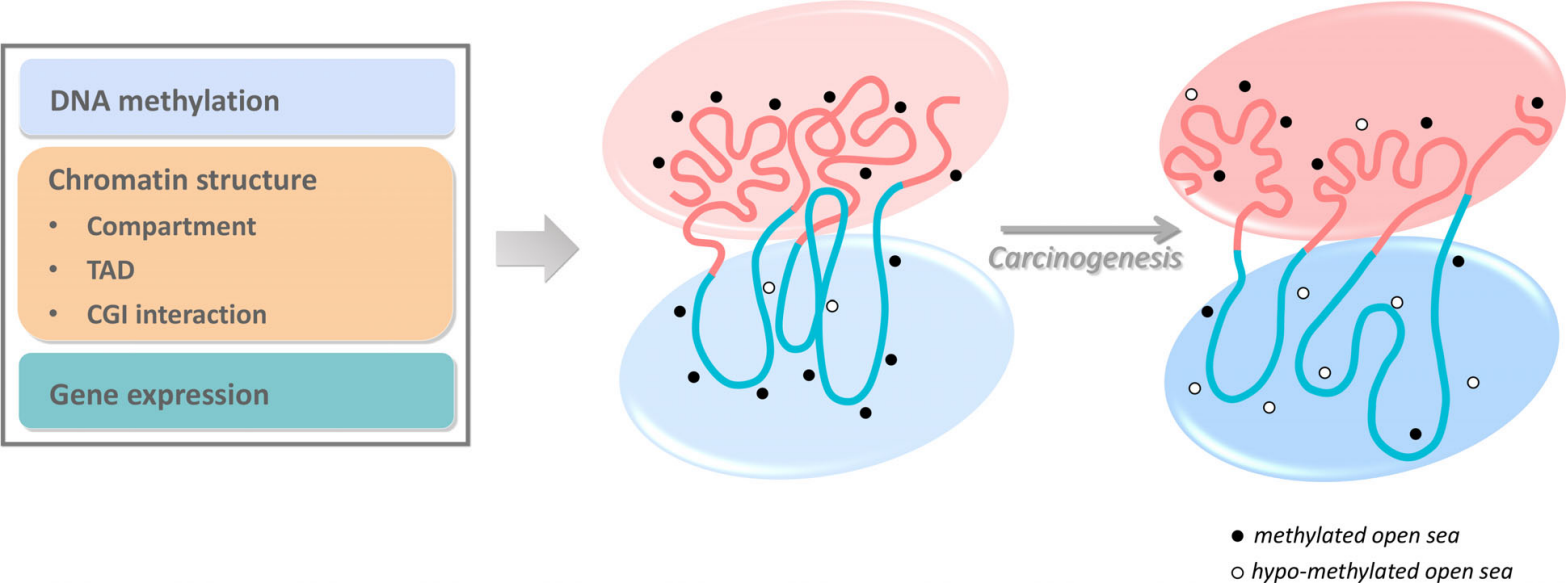
Concerted changes in DNA methylation, chromatin structure, and gene expression in carcinogenesis. Forest and prairie domains are represented by pink and blue lines, respectively. The pink circle represents a more open and active state, whereas the blue circle represents a relative repressive state. The differences between the forests and prairies are enlarged in carcinogenesis. Small black circles show the methylation states of open seas, to be specific, filled and hollow ones mean methylated and hypomethylated open seas.

In the present study, we performed an integrated analysis of DNA methylation, 3D chromatin structure, DNase hypersensitivity, and gene expression (Fig. 6). We found several common trends that are associated with carcinogenesis in various cancer types: (a) a consistent global chromatin structure change in which the short genomic distance contacts increase in the expense of long distance contacts, especially at Mb scale. (b) Enhanced separation of genome segments of different CpG densities at the scales of both CGI (kb) and CGI forests/prairies (Mb). Domains of similar CpG density and methylation level tend to gain contacts. (c) The loss of the contacts of low CpG prairie domains with the CpG rich domains coincides with their hypomethylation and gives rise to a larger difference between the open seas in the forest and prairie domains, which is aggravated as cancer stage increases. (d) The expression‐level difference between the more active CGI/forests and less active non‐CGI/prairies is enlarged in cancer cells, compared to normal samples. These observations suggest that in cancer development, chromatin goes through concerted structure, epigenetics, and expression activity changes that are strongly influenced by sequential properties.

In general, it is believed that cancer is driven by genetic change and a set of driver mutations are identified. However, 5% of cancer cases had no drivers that can be identified in a recent work [70]. One possible explanation is that mutations are not the only factor initiating and promoting the cancer development, common epigenetic changes maybe correlated with carcinogenesis. Since there is a case that almost no methylation changes are observed in CGI and open sea at very early stages of cancer (luad5, Fig. 1A), it is tempting to speculate that methylation changes might not be the earliest changes in all carcinogenesis either. We should also notice that the different extents of methylation changes in different cancer stages might be influenced by the experiment bias and that the early stage samples may consist of less cancer cells but more normal cells compared with the late stage samples. The methylation changes at early tumor stage remain to be investigated.

Notably, our analysis of Hi‐C data shows that chromatin 3D structure is an important variable that closely correlate with cell state, which could be used to distinguish the tumor from normal cells we examined by clustering analysis (Fig. 2C). The significant structure changes identified also show potential relationship with key cancer properties, such as cell division, adhesion, and immune response. Therefore, the relationship between the establishment and destruction of well‐organized chromatin structure and carcinogenesis is worthy of further exploration.

It appears that genomic sequence itself is one determining factor in the formation of high‐order structure. As responses to the cellular environment, structural modifiers, such as TFs, miRNA, DNA methyltransferase and histone modifiers, all contribute to the formation of specific chromosome structures to achieve cell identity and cell function. In cancer cell lines, for the overall chromosomal structure, the enhanced domain segregation between forests and prairies is likely driven by the aggregation of prairies, consistent with the finding that attractions between heterochromatic regions are crucial for the formation of compartments [71], and facilitated by the large number of cell cycles the cells experienced. From the perspective of CGI, during carcinogenesis, CGIs within the same genome type (forest or prairie) tend to aggregate. Such conservative structural changes are found to correlate with functions corresponding to carcinogenesis and cancer development. However, the mechanisms of these changes are not clear and the gene regulatory networks in cancer need to be further investigated. We speculate that since many transcription factors bind CGI‐rich regions, the higher spatial contacts within CGIs may provide an open and active environment for related genes’ transcription in cancer (e.g., through a liquid–liquid phase separation mechanism [72]).

## 5 Conclusions

In summary, we found consistent enlarged structural, epigenetic, and expression differences between forests and prairies in various types of cancer cells. Although the causal relationship between them needs to be clarified, the general sequence dependence of various genomic and epigenetic changes provides us with a new perspective and the possibility of a more general mechanism of carcinogenesis. The difference among primary cancer, leukemia, and cancer cell line needs to be further investigated in the future. We hope such knowledge will eventually help us develop novel cancer diagnostic and therapeutic methods.

## Conflict of interest

The authors declare no conflict of interest.

### Peer review

The peer review history for this article is available at https://publons.com/publon/10.1002/1878‐0261.13127.

## Author contributions

YQG conceptualized the study; YX, YY, HT, SL, LZ, LY, and HZ curated the data; YX, YY, HT, HQ, and SL involved in formal analysis; YQG and LY involved in funding acquisition; YX, YY, HT, HQ, SL, LZ, LY, HZ, HW, and YQG investigated the study; YQG supervised the study; YX visualized the study; YX, YY, HT, HQ, and SL wrote—original draft; HW and YQG wrote—review & editing. All authors have read and agreed to the published version of the manuscript.

## Supporting information


**Fig**. **S1**. Methylation changes in carcinogenesis.
**Fig**. **S2**. Methylation changes for open sea in carcinogenesis.
**Fig**. **S3**. General chromatin architecture in cancer cell lines.
**Fig**. **S4**. Chromatin structure changes in carcinogenesis.
**Fig**. **S5**. CGI aggregation in carcinogenesis.
**Fig**. **S6**. Average DNase signal at various methylation levels.
**Fig**. **S7**. Validation of the correlations between DNA methylation and accessibility.
**Fig**. **S8**. The methylation differences between forest and prairie.
**Fig**. **S9**. Gene expression in cancer cells.

**Table S1**. Data information.
**Table S2**. The genome coordinates of F and P.

**Table S3**. The proportion of compartment B in normal and tumorous samples (chr1 to chr22).
**Table S4**. Averaged compartment vector in regions with different sequential properties (chr1).
**Table S5**. The proportion of functional genes undergo F‐F contact break (600K‐2 M) in carcinogenesis.
**Table S6**. GO enrichment analysis of aggregated F‐CGI genes and P‐CGI genes with increased expression in A549, Panc1 and HepG2.

## Data Availability

The data that support the findings of this study were derived from the following resources available in the public domain: TCGA, ENCODE, Roadmap, and GEO. The detailed data accession can be found in Table S1.
