# Supplementary material for: Computational characterization of domain‐segregated 3D chromatin structure and segmented DNA methylation status in carcinogenesis
Source: Mol Oncol. 2021 Nov 9;16(3):699–716. doi: 10.1002/1878-0261.13127 (PMC8807360; doi:10.1002/1878-0261.13127)
Supplement: Supplementary file 1 — Fig. S1. Methylation changes in carcinogenesis. Fig. S2. Methylation changes for open sea in carcinogenesis. Fig. S3. General chromatin architecture in cancer cell lines. Fig. S4. Chromatin structure changes in carcinogenesis. Fig. S5. CGI aggregation in carcinogenesis. Fig. S6. Average DNase signal at various methylation levels. Fig. S7. Validation of the correlations between DNA methylation and accessibility. Fig. S8. The methylation differences between forest and prairie. Fig. S9. Gene expression in cancer cells. [file MOL2-16-699-s001.pdf]

Computational characterization of domain-segregated 3D chromatin structure and segmented DNA methylation status in carcinogenesis

Yue Xue[1], Ying Yang[1], Hao Tian[1], Hui Quan[1], Sirui Liu[1], Ling Zhang[1], Lu Yang[2,3], Haichuan Zhu[2,3], Hong Wu[2,3,4] and Yi Qin Gao[1,5,6,*]

1 Beijing National Laboratory for Molecular Sciences, College of Chemistry and Molecular Engineering, Peking University, Beijing 100871, China
2 The MOE Key Laboratory of Cell Proliferation and Differentiation, School of Life Sciences, Peking University, Beijing 100871, China
3 Peking-Tsinghua Center for Life Sciences, Peking University, Beijing 100871, China
4 Peking University Institute of Hematology, National Clinical Research Center for Hematologic Disease, Peking University People's Hospital, Beijing 100044, China
5 Biomedical Pioneering Innovation Center (BIOPIC), Peking University Beijing, 100871, China
6 Beijing Advanced Innovation Center for Genomics (ICG), Peking University, Beijing 100871, China

* Correspondence: gaoyq@pku.edu.cn; Tel: +86-10-62752431

I Supplementary Figures S1-S9

II Supplementary Tables S1, S3-S5

Supplementary Figures

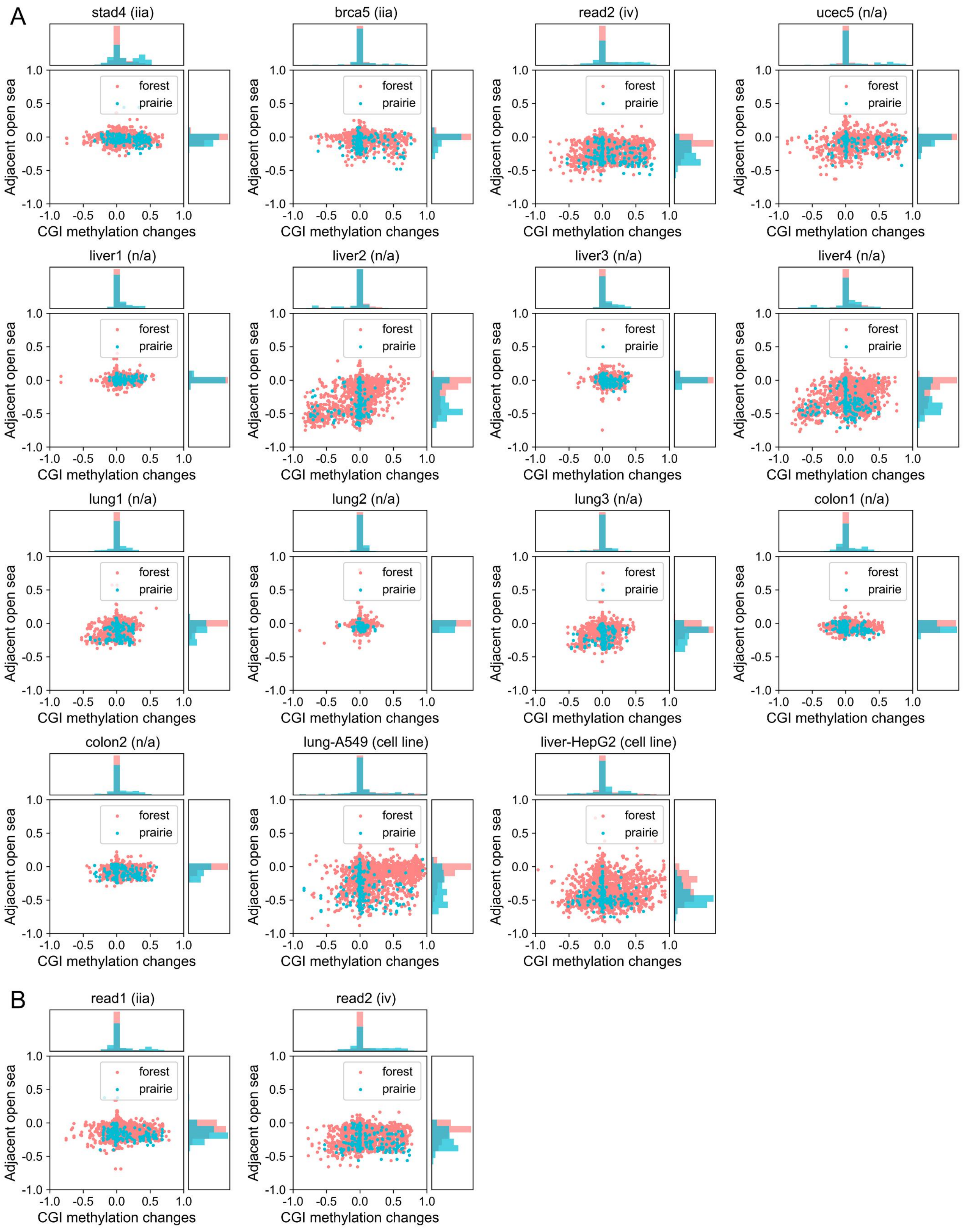

Fig. S1. Methylation changes in carcinogenesis. (**A**) Scatter plots for changes of methylation level in CGIs and open seas. Each dot represents the methylation changes of a CGI (x axis) and its adjacent

open sea (y axis) compared between adjacent normal samples and their corresponding cancer samples. The probability density distribution of CGI and open sea methylation changes are shown on the top and right sides of the figure, respectively. The cancer stages are given next to the sample names. (**B**) Methylation changes for two rectum adenocarcinoma samples at different cancer stages. read1 and read2 both use read_n2 as normal control because the former doesn't have adjacent normal tissue data.

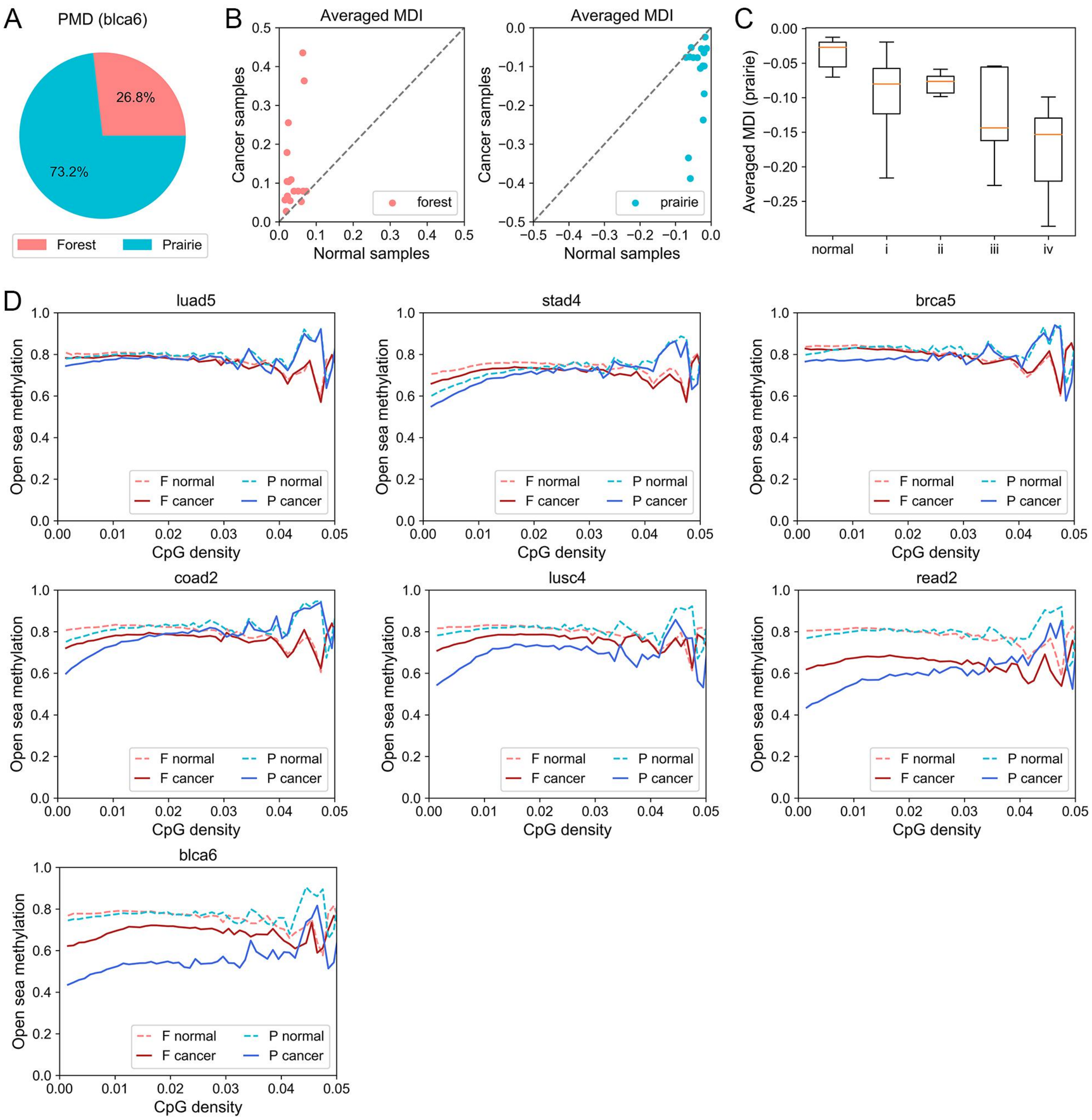

Fig. S2. Methylation changes for open sea in carcinogenesis. (**A**) The sequence composition of PMD in blca6 cancer sample. Among all cancer samples, 72.9% of PMDs are located in prairie on average. (**B**) Averaged MDIs for forests and prairies in normal and cancer samples. Each dot represents one pair of samples. (**C**) The averaged MGIs of all prairie domains in normal samples and cancer samples at different stages. (**D**) CpG density and methylation level are calculated for all 1-kb beads in open sea. Each point on the curve shows the averaged methylation level for beads possess a given CpG density.

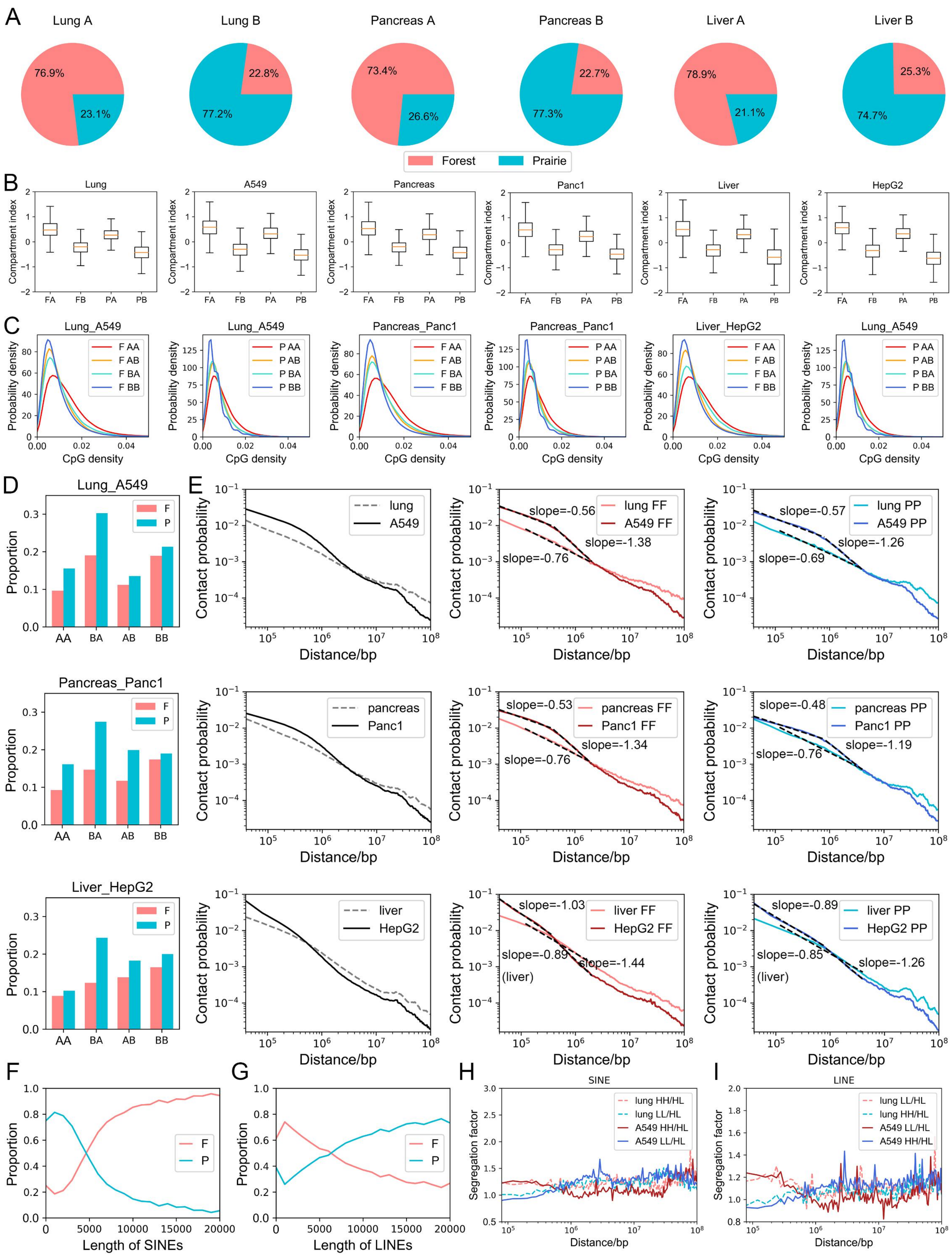
A
Lung A
Lung B
Pancreas A
Pancreas B
Liver A
Liver B
76.9%
23.1%
22.8%
77.2%
73.4%
26.6%
22.7%
77.3%
78.9%
21.1%
25.3%
74.7%
Forest
Prairie
B
Lung
A549
Pancreas
Panc1
Liver
HepG2
Compartment index
FA
FB
PA
PB
C
Lung_A549
Pancreas_Panc1
Liver_HepG2
F AA
F AB
F BA
F BB
P AA
P AB
P BA
P BB
Probability density
CpG density
D
Lung_A549
Pancreas_Panc1
Liver_HepG2
Proportion
F
P
AA
BA
AB
BB
E
lung
A549
lung FF
A549 FF
lung PP
A549 PP
slope=-0.56
slope=-1.38
slope=-0.76
slope=-0.57
slope=-1.26
slope=-0.69
pancreas
Panc1
pancreas FF
Panc1 FF
pancreas PP
Panc1 PP
slope=-0.53
slope=-1.34
slope=-0.76
slope=-0.48
slope=-1.19
slope=-0.76
liver
HepG2
liver FF
HepG2 FF
liver PP
HepG2 PP
slope=-1.03
slope=-0.89
(liver)
slope=-1.44
slope=-0.89
slope=-0.85
(liver)
slope=-1.26
Contact probability
Distance/bp
F
Length of SINEs
G
Length of LINEs
H
SINE
lung HH/HL
lung LL/HL
A549 HH/HL
A549 LL/HL
Segregation factor
I
LINE
lung LL/HL
lung HH/HL
A549 LL/HL
A549 HH/HL

Fig. S3. General chromatin architecture in cancer cell lines. (**A**) The proportion of forest and prairie sequences in compartments A and B for lung, pancreas and liver. (**B**) The compartment index for forest domains in compartment A (FA) and compartment B (FB), prairie domains in compartment A (PA) and compartment B (PB) in lung, A549, pancreas, Panc1, liver and HepG2. (**C**) The probability density of CpG density for forest and prairie domains which belong to conservative compartment A (F AA) or B (F BB), as well as domains switch from A to B (F AB) or B to A (F BA) in carcinogenesis, *P*-values $< 10^{-300}$ by Welch's unequal variance t-test between the CpG density of AA and AB. (**D**) The proportion of complementary tissue-specific genes for genes located in AA, AB, BA and BB in carcinogenesis. (**E**) The contact probability at varied genomic distances (left column, chromosome 1 is used as an example), the contact probability between forests (middle column) and between prairies (right column) in lung and A549, pancreas and Panc1, liver and HepG2. (**F**) The proportion of F- and P-domains for 40-kb bins with different lengths of SINEs and (**G**) LINEs. (**H**) The segregation factor between bins with high density (H) and low density (L) of SINEs at varied genomic distances on chromosome 1 for lung and A549. (**I**) The segregation factor between bins with high density (H) and low density (L) of SINEs at varied genomic distances on chromosome 1 for lung and A549.

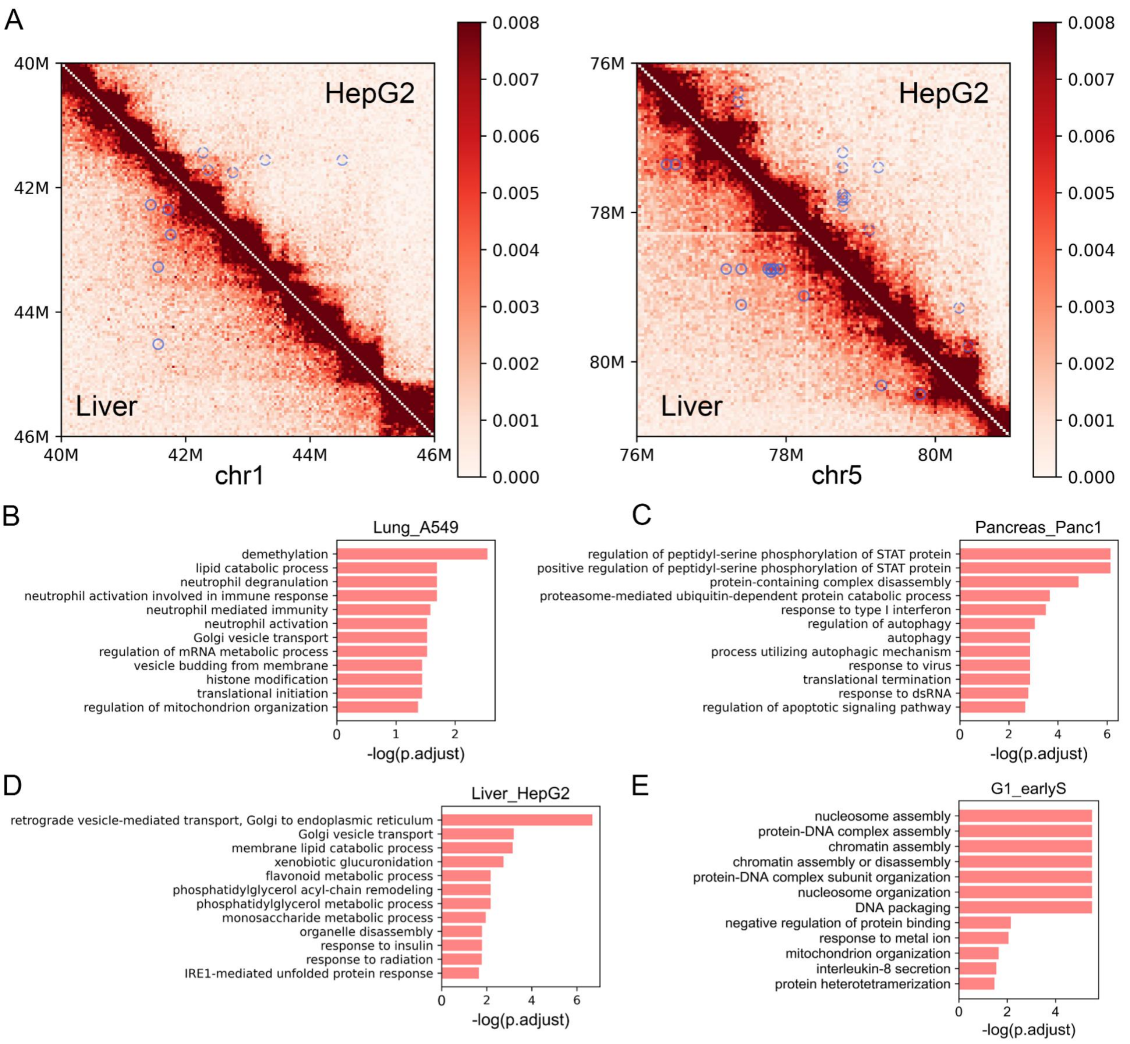

Fig. S4. Chromatin structure changes in carcinogenesis. (**A**) The contact probability matrix of liver (lower triangular matrix) and HepG2 (upper triangular matrix) (chromosome 1, within a F-domain). Contacts within blue circles are the chromatin interactions detected by Fit-Hi-C in liver but not in HepG2, which also lose contacts with F-domains from liver to HepG2. (**B**) GO analysis for forest genes which loss contact with other forest domains at 600K to 2M from lung to A549, (**C**) from pancreas to Panc1, (**D**) from liver to HepG2 and (**E**) from G1 to early S stage in mouse cell cycle.

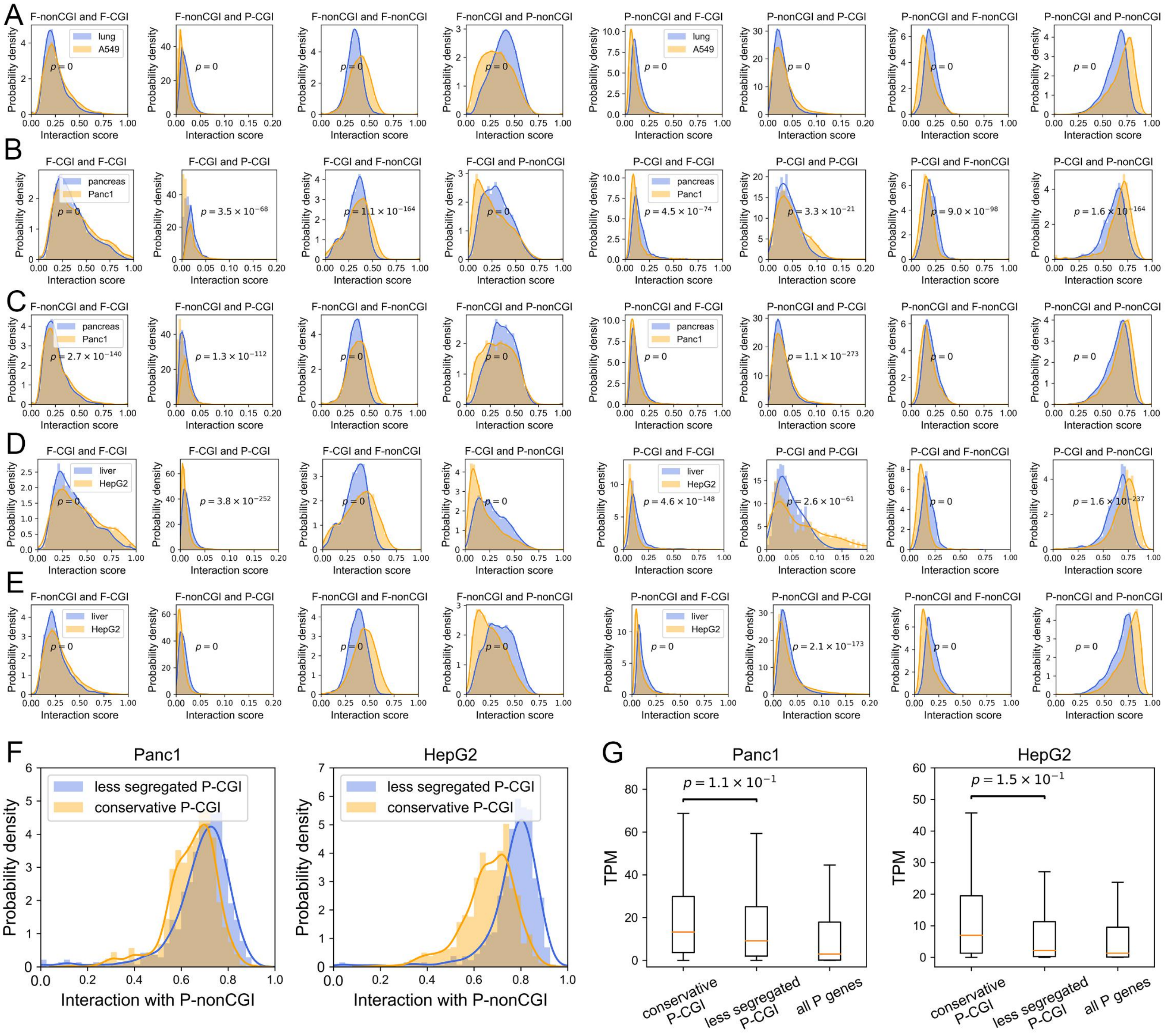

Fig. S5. CGI aggregation in carcinogenesis. (**A**) The interaction scores between F-nonCGI (top) or P-nonCGI (bottom) and the four types of domains (F-CGI, F-nonCGI, P-CGI, and P-nonCGI) in normal lung and A549. Interaction scores for (**B**) F-CGI, P-CGI and (**C**) F-nonCGI, P-nonCGI in normal pancreas and Panc1. Interaction scores for (**D**) F-CGI, P-CGI and (**E**) F-nonCGI, P-nonCGI in normal liver and HepG2. The *P*-values calculated by Welch's unequal variance t-test are shown on the figure and p=0 means that *P*-value < 10^{-300}. (**F**) The probability density of interactions with P-nonCGI for conservative P-CGIs and P-CGIs becoming less segregated in Panc1 and HepG2. (**G**) The expression level for conservative P-CGI genes, less segregated P-CGI genes and all prairie genes in Panc1 and HepG2. Expression level for each gene is calculated by averaging TPM over all PAAD cancer samples and LIHC cancer samples, respectively. P-values are calculated by Welch's unequal variance t-test.

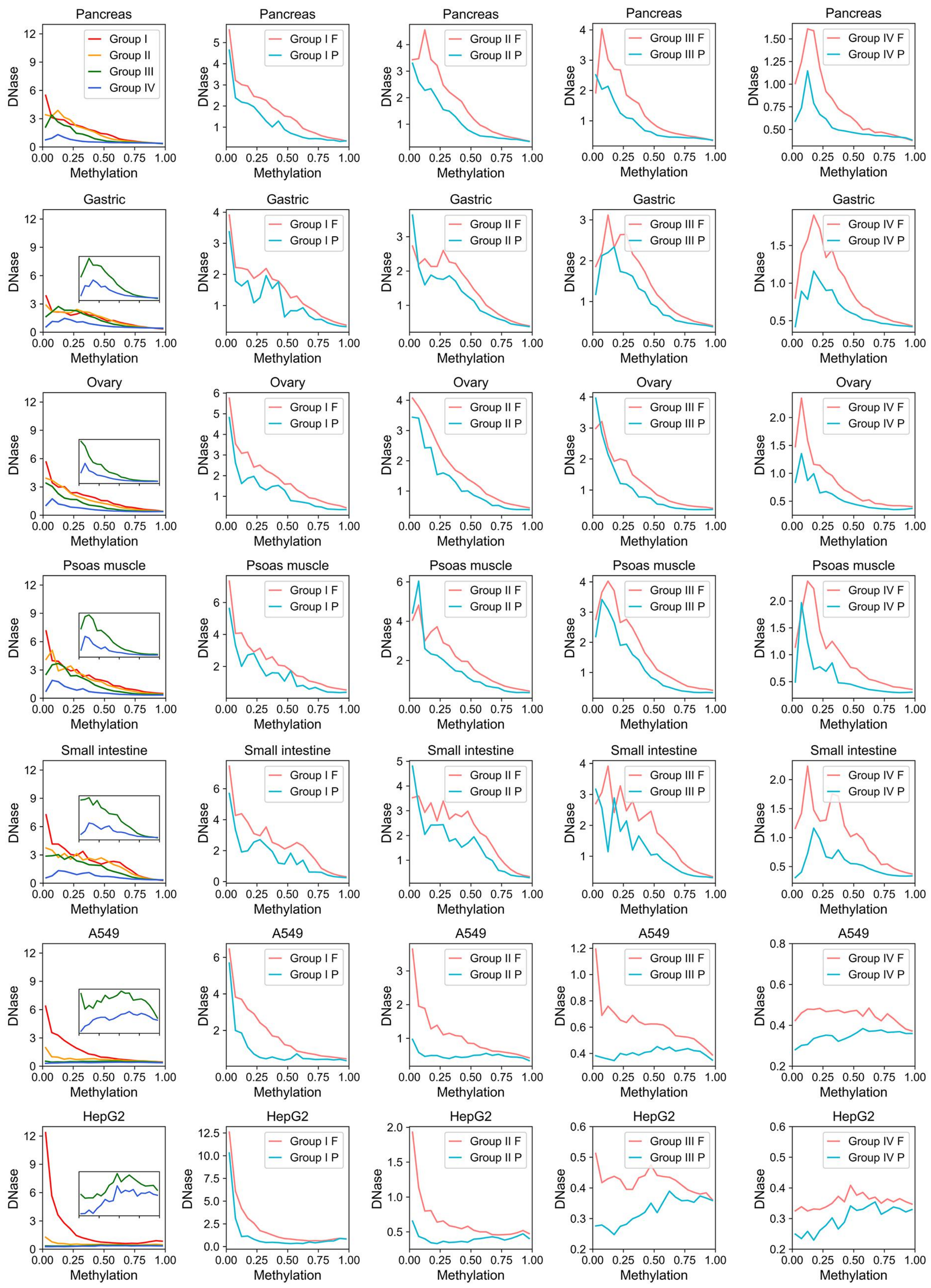

Fig. S6. Average DNase signal at various methylation levels. CpG density and CpG methylation level are calculated at a 1-kb resolution. Bins are divided to groups I, II, III or IV according to their CpG density (2.0%, 20.1%], (1.0%, 2.0%], (0.5%, 1.0%] or (0, 0.5%], respectively.

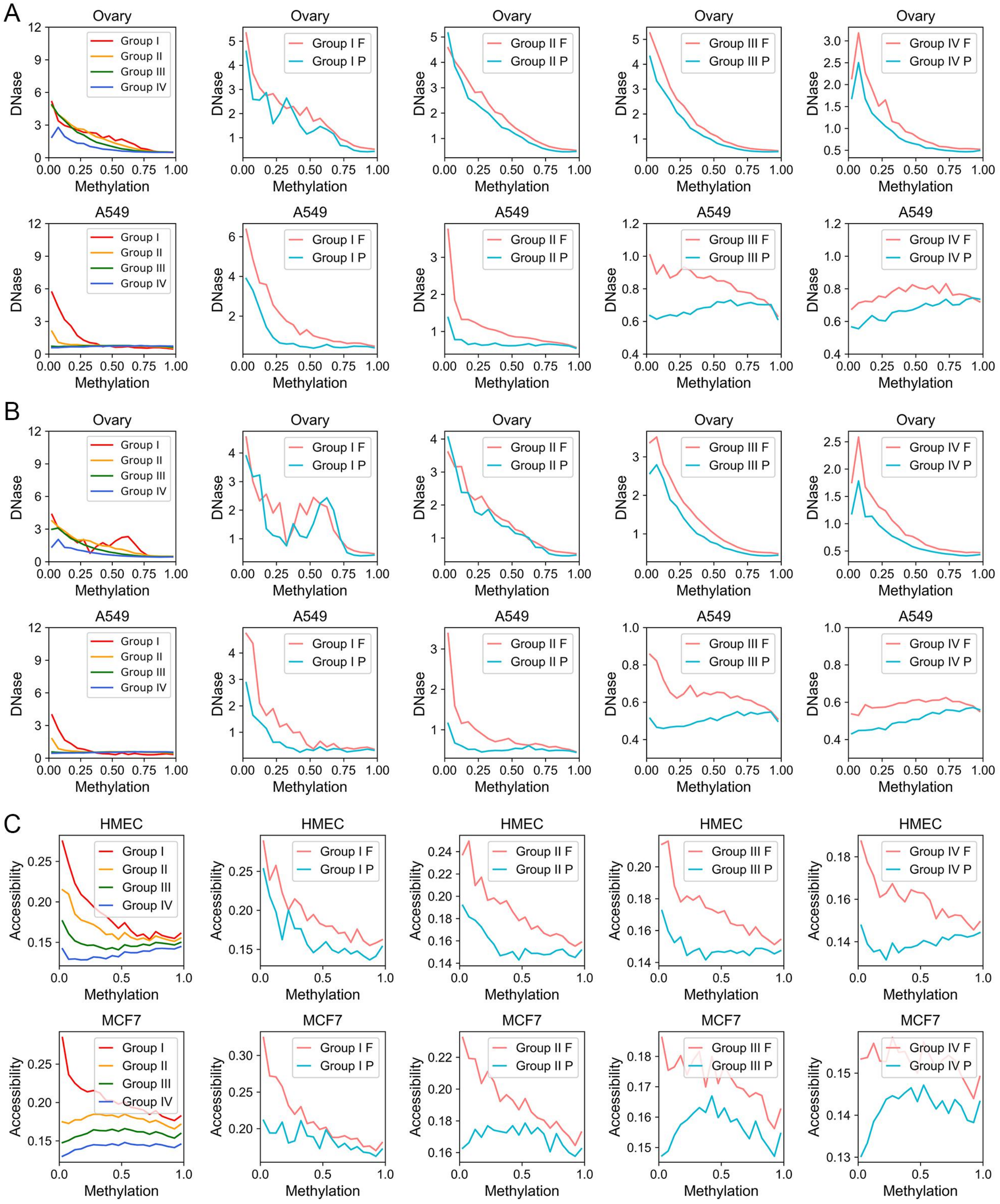

Fig. S7. Validation of the correlations between DNA methylation and accessibility. For 1-kb bins whose GC content belong to (**A**) (0.4, 0.45] and (**B**) (0.35, 0.4], average DNase signal at various methylation levels for bins with different CpG density group. (**C**) DNA methylation and accessibility (whether occupied by nucleosomes) acquired from NoMe-seq data for normal human mammary epithelial cells (HMEC) and breast cancer cell line (MCF7).

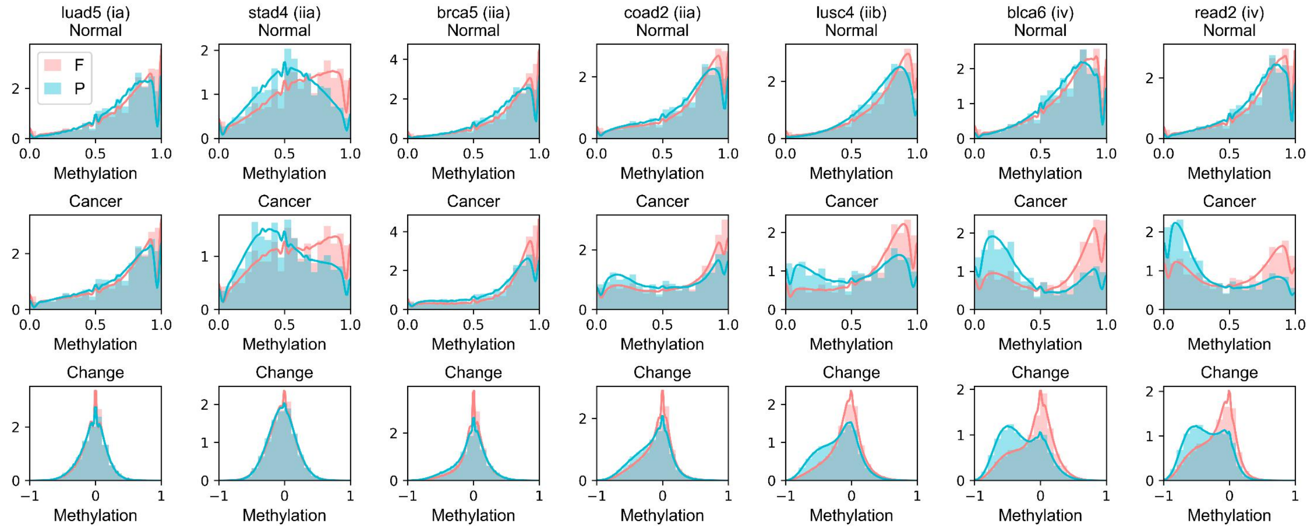

Fig. S8. The methylation differences between forest and prairie. The probability density of methylation level of solo-WCGW. ‘solo’ refers to the CpGs with no neighboring CpGs and ‘W’ indicates A or T nucleotide. Each row is one sample. The methylation in normal tissue, cancer sample and changes are shown on the top, middle and bottom line, respectively.

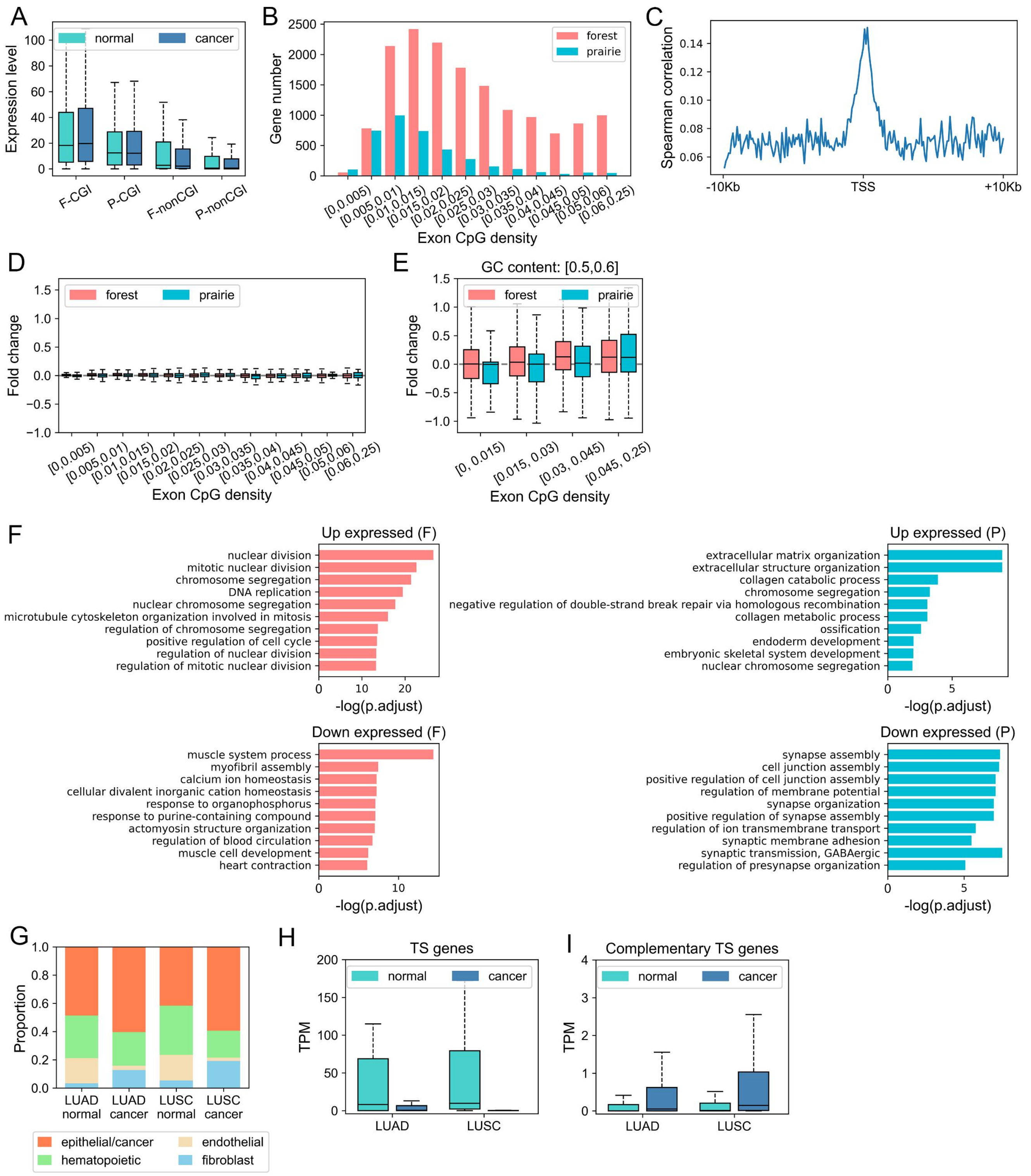

Fig. S9. Gene expression in cancer cells. (**A**) Boxplots for the average expression levels (TPM) for F-CGI genes, P-CGI genes, F-nonCGI genes and P-nonCGI genes in all normal and cancer samples. (**B**) The amount of genes with different exon CpG density. (**C**) The Spearman correlation between expression fold change in carcinogenesis and CpG density (in 100 bp resolution) around TSSs for coding genes. (**D**) Normal samples are randomly divided to two groups. The boxplot shows the

expression fold difference between the two normal groups as a control of Fig. 5F. (**E**) The expression fold changes in carcinogenesis (see methods) for genes whose exon GC content belongs to [0.5, 0.6] but with various exon CpG density. (**F**) GO analysis for commonly differentially expressed genes. (**G**) The proportion of epithelial/cancer, endothelial, hematopoietic and fibroblast cells estimated by CIBERSORTx from matched normal and cancer RNA-seq data of LUAD and LUSC. (**H**) The expression level of tissue-specific genes and (**I**) complementary tissue-specific genes in epithelial/cancer-specific subpopulation inferred by CIBERSORTx from normal and cancer samples.

Supplemental Tables

Table S1. Data information.

Data type	Abbreviation	Cell type	Cell state	Sample ID or accession	Data source
WGBS	blca_t1	Bladder urothelial carcinoma	cancer stage iii	TCGA-DK-A1AA-01A-11D-A23D-05	TCGA
	blca_t2		cancer stage iii	TCGA-DK-A1AG-01A-11D-A23D-05	
	blca_t3		cancer stage iv	TCGA-BL-A13J-01A-11D-A23D-05	
	blca_t4		cancer stage iii	TCGA-BT-A2LA-01A-11D-A23D-05	
	blca_t5		cancer stage iv	TCGA-H4-A2HQ-01A-11D-A23D-05	
	blca_t6		cancer stage iv	TCGA-BT-A20V-01A-11D-A23D-05	
	blca_n6		normal	TCGA-BT-A20V-11A-11D-A23D-05	
	brca_t1	Breast invasive carcinoma	cancer stage iia	TCGA-A2-A04X-01A-21D-A19F-05	
	brca_t2		cancer stage iiia	TCGA-A8-A07I-01A-11D-A19F-05	
	brca_t3		cancer stage iiic	TCGA-A2-A0YG-01A-21D-A19F-05	
	brca_t4		cancer stage iia	TCGA-E2-A15H-01A-11D-A19F-05	
	brca_t5		cancer stage iia	TCGA-A7-A0CE-01A-11D-A148-05	
	brca_n5		normal	TCGA-A7-A0CE-11A-21D-A148-05	
	coad_t1	Colon adenocarcinoma	cancer stage i	TCGA-AA-A00R-01A-01D-A22T-05	
	coad_t2		cancer stage iia	TCGA-AA-3518-01A-02D-1518-05	
	coad_n2		normal	TCGA-AA-3518-11A-01D-1518-05	
	gbm_t1	Glioblastoma multiforme	cancer	TCGA-06-0128-01A-01D-2294-05	
	gbm_t2		cancer	TCGA-14-1454-01A-01D-2294-05	
	gbm_t3		cancer	TCGA-14-3477-01A-01D-2294-05	
	gbm_t4		cancer	TCGA-14-1401-01A-01D-2294-05	
	gbm_t5		cancer	TCGA-16-1460-01A-01D-2294-05	
	gbm_t6		cancer	TCGA-19-1788-01A-01D-2294-05	
	luad_t1	Lung adenocarcinoma	cancer stage ib	TCGA-38-4630-01A-01D-2365-05	
	luad_t2		cancer stage ib	TCGA-67-6215-01A-11D-2365-05	
	luad_t3		cancer stage iv	TCGA-78-7156-01A-11D-2365-05	
	luad_t4		cancer stage ia	TCGA-91-6840-01A-11D-2365-05	

	luad_t5		cancer stage ia	TCGA-44-6148-01A-11D-2365-05	
	luad_n5		normal	TCGA-44-6148-11A-01D-2365-05	
	lusc_t1	Lung squamous cell carcinoma	cancer stage ia	TCGA-34-2600-01A-01D-1871-05	
	lusc_t2		cancer stage ib	TCGA-60-2695-01A-01D-1871-05	
	lusc_t3		cancer stage ib	TCGA-21-1078-01A-01D-2365-05	
	lusc_t4		cancer stage iib	TCGA-60-2722-01A-01D-1871-05	
	lusc_n4		normal	TCGA-60-2722-11A-01D-1871-05	
	stad_t1	Stomach adenocarcinoma	cancer stage iv	TCGA-CG-5730-01A-11D-2365-05	
	stad_t2		cancer	TCGA-D7-6519-01A-11D-2365-05	
	stad_t3		cancer stage i	TCGA-F1-6177-01A-11D-2365-05	
	stad_t4		cancer stage iia	TCGA-BR-6452-01A-12D-2365-05	
	stad_n4		normal	TCGA-BR-6452-11A-01D-2365-05	
	read_t1	Rectum adenocarcinoma	cancer stage iia	TCGA-AG-3593-01A-01D-2294-05	
	read_t2		cancer stage iv	TCGA-AF-2689-01A-01D-2294-05	
	read_n2		normal	TCGA-AF-2689-11A-01D-2294-05	
	ucec_t1	Uterine corpus endometrial carcinoma	cancer	TCGA-B5-A0K6-01A-11D-A23D-05	
	ucec_t2		cancer	TCGA-AX-A1CK-01A-11D-A23D-05	
	ucec_t3		cancer	TCGA-AP-A05J-01A-11D-A23D-05	
	ucec_t4		cancer	TCGA-A5-A0G2-01A-11D-A23D-05	
	ucec_t5		cancer	TCGA-AX-A1CI-01A-11D-A17H-05	
	ucec_n5		normal	TCGA-AX-A1CI-11A-11D-A17H-05	
	liver_t1	Liver tumor and adjacent normal tissue	cancer	GSE70090	Ref. (28)
	liver_t2		cancer		
	liver_t3		cancer		
	liver_t4		cancer		
	liver_n1		normal		
	liver_n2		normal		
	liver_n3		normal		
	liver_n4		normal		
	lung_t1	Lung tumor and	cancer		

	lung_t2	adjacent normal tissue	cancer		
	lung_t3		cancer		
	lung_n1		normal		
	lung_n2		normal		
	lung_n3		normal		
	colon_t1	colon moderately differentiated adenocarcinoma and adjacent normal tissue	cancer	GSE46644	Ref. (29)
	colon_n1		normal		
	colon_t2	Colorectal cancer and adjacent normal tissue	cancer	GSE52271	Ref. (30)
	colon_n2		normal		
	lung	lung (donor stl002)	normal	GSM983647	GEO
	A549	lung cancer	cancer cell line	GSE127301	
	liver	liver (donor 3)	normal	GSM916049	
	HepG2	liver cancer	cancer cell line	GSE46644	
Hi-C	cortex	cortex	brain normal cell	GSE87112	Ref. (37)
	hippocampus	hippocampus			
	adrenal	adrenal	somatic normal cell		
	aorta	aorta			
	bladder	bladder			
	left_ventricle	left ventricle			
	liver	liver			
	lung_1	lung			
	lung_2	lung			
	ovary	ovary			
	pancreas_2	pancreas			
	pancreas_3	pancreas			
	psoas_1	psoas			
	psoas_3	psoas			
	right_ventricle	right ventricle			

	small_bowel	small bowel			
	spleen_1	spleen			
	spleen_3	spleen			
	H1	embryonic stem cell	normal		
	A549_1	lung cancer	cancer cell line	ENCSR444WCZ	ENCODE
	A549_2				
	Caki2_1	kidney cancer		ENCSR401TBQ	
	Caki2_2				
	G401_1	wilms tumor		ENCSR079VIJ	
	G401_2				
	LNCaP_1	prostate cancer		ENCSR346DCU	
	LNCaP_2				
	NCI-H460_1	lung cancer		ENCSR489OCU	
	NCI-H460_2				
	Panc1_1	pancreatic cancer		ENCSR440CTR	
	Panc1_2				
	RPMI-7951_1	melonoma		ENCSR862OGI	
	RPMI-7951_2				
	SK-MEL-5_1	melonoma		ENCSR312KHQ	
	SK-MEL-5_2				
	SK-N-DZ_1	neuroblastoma		ENCSR105KFX	
	SK-N-DZ_2				
	SK-N-MC_1	askins tumor		ENCSR834DXR	
	SK-N-MC_2				
	T47D_1	breast cancer		ENCSR549MGQ	
	T47D_2				
	HepG2	liver cancer		ENCSR194SRI	
	growing	growing cell	normal	PRJEB8073	Ref. (36)
	senescence	senescent cell	normal		
	G1	G1 stage of mouse	normal	GSE94489	Ref. (40)

		embryonic stem cell			
	early S	early S stage of mouse embryonic stem cell	normal		
	normal1	peripheral blood	normal	GSE146901	Ref. (38)
	normal2				
	normal3				
	normal4				
	32	T-lineage acute lymphoblastic leukemia (T-ALL)	leukemia		
	76				
	77				
	93				
	97				
	98				
	102				
	103				
	107				
	108				
	115				
	116				
	117				
	118				
	121				
	122				
	123				
	124				
NoMe-seq	HMEC	normal human mammary epithelial cells	cell line	GSE57498 (https://zenodo.org/record/12454)	Ref. (61)
	MCF7	breast cancer cell line	cancer cell line		

Table S3. The proportion of compartment B in normal and tumorous samples (chr1 to chr22).

	Normal sample	Tumorous cell line
Pancreas	0.5222	0.6205
Lung	0.5548	0.5718
Liver	0.5964	0.5844

Table S4. Averaged compartment vector $\overline{V}$ in regions with different sequential properties (chr1).

			Normal sample	Tumorous cell line	Difference *
Lung	forest	CGI	0.0205	0.0159	-0.0046
		nonCGI	0.0089	0.0058	-0.0031
		Difference†	0.0115	0.01	-
	prairie	CGI	-0.0183	-0.0256	-0.0073
		nonCGI	-0.0247	-0.0269	-0.0022
		Difference	0.0064	0.0013	-
Pancreas	forest	CGI	0.0236	0.0126	-0.011
		nonCGI	0.013	0.001	-0.012
		Difference	0.0106	0.0116	-
	prairie	CGI	-0.0091	-0.0308	-0.0218
		nonCGI	-0.0133	-0.0283	-0.015
		Difference	0.0042	-0.0025	-
Liver	forest	CGI	0.0183	0.0184	0.0001
		nonCGI	0.0087	0.0074	-0.0013
		Difference	0.0096	0.0110	-
	prairie	CGI	-0.0191	-0.0204	-0.0013
		nonCGI	-0.0235	-0.0260	-0.0025
		Difference	0.0044	0.0056	-

* The sample difference is defined as the averaged compartment vector $\overline{V}$ of tumorous cell line minus that of normal sample in the same region.

† The region difference is defined as the $\overline{V}$ of CGI regions minus that of nonCGI regions, and is calculated for forest and prairie respectively.

Table S5. The proportion of functional genes undergo F-F contact break (600K-2M) in carcinogenesis.

GO function	Hi-C sample	F genes which loss F contact	All F functional genes in Hi-C *	F functional genes which loss F contact	Proportion of FF loss genes †	Proportion of functional genes
Antigen processing presentation	lung_A549	3304	152	45	1.36%	29.61%
	pancreas_Panc 1	3247	147	41	1.26%	27.89%
	liver_HepG2	3369	160	39	1.16%	22.50%
	common	878		16	1.82%	
Immune system	LG2_LGt	3304	1868	489	14.80%	26.18%
	PA2_Panc1	3247	1803	497	15.31%	27.57%
	liver_HepG2	3369	1899	471	14.0%	24.80%
	common	878		117	13.3%	

* The number of F immune genes which are involved in F-F contact calculation in given Hi-C matrix.

† For comparison, F antigen genes and F immune system genes account for 1.26% and 13.91% of F genes, respectively.
